# Tropical Cyclone Exposure and Psychoactive Drug–Related Death Rates

**DOI:** 10.1001/jamanetworkopen.2025.60183

**Published:** 2026-02-20

**Authors:** Raenita Spriggs, Victoria D. Lynch, Yuanyu Lu, Lincole Jiang, Wil Lieberman-Cribbin, G. Brooke Anderson, Katherine M. Keyes, Marianthi-Anna Kioumourtzoglou, Diana Hernández, Anne E. Nigra, Robbie M. Parks

**Affiliations:** 1 Department of Environmental Health Sciences, Mailman School of Public Health, Columbia University, New York, New York; 2 Department of Biostatistics, Mailman School of Public Health, Columbia University, New York, New York; 3 Department of Occupational Medicine, Prevention, and Epidemiology, Northwell Health, Great Neck, New York; 4 Department of Environmental and Radiological Health Sciences, College of Veterinary Medicine and Biomedical Sciences, Colorado State University, Fort Collins, Colorado; 5 Department of Epidemiology, Mailman School of Public Health, Columbia University, New York, New York; 6 Department of Epidemiology, Brown School of Public Health, Brown University, Providence, Rhode Island; 7 Institute at Brown University for Environment and Society, Brown University, Providence, Rhode Island; 8 Department of Sociomedical Sciences, Mailman School of Public Health, Columbia University, New York, New York

## Abstract

**Importance:**

Tropical cyclone exposure exacerbates substance use and mental and behavioral health vulnerabilities in affected communities, potentially contributing to increases in psychoactive drug–related deaths (including drug overdoses, alcohol-related deaths, and other substance-related disorders). Prior research has not evaluated this association in a national multidecadal study by cyclone strength and demographic subgroup.

**Objective:**

To estimate the association between county-level tropical cyclone exposure and psychoactive drug–related death rates in the US.

**Design, Setting, and Participants:**

This case-control study used a bayesian conditional quasi-Poisson model to evaluate the association between county-level tropical cyclone exposure and monthly psychoactive drug–related death rates between January 1, 1988, and December 31, 2019. Participants included residents of counties in the contiguous US that were exposed to at least 1 tropical cyclone during the study period. Death data were obtained from the National Center for Health Statistics. Analyses were conducted between March 27, 2024, and June 25, 2025.

**Exposure:**

Monthly tropical cyclone–exposed days per county, defined by sustained wind speeds of at least 34 knots, categorized as gale to violent storms (34-63 knots) and hurricanes (≥64 knots).

**Main Outcomes and Measures:**

The main outcome was monthly county-level psychoactive drug–related death rates. Associations were estimated between each additional cyclone-exposed day and death rates in the subsequent 0 to 3 months. Analyses were stratified by cyclone strength, age, sex, and county-level social disadvantage (poverty level and percentage of racial and ethnic minority residents).

**Results:**

Among 1258 exposed counties between 1988 and 2019, 798 691 psychoactive drug–related deaths were recorded (29.5% females and 70.5% males; 99.8% aged ≥15 years). Median exposure was 2 tropical cyclone days (range, 1-27 days) during the study. Each additional cyclone-exposed day was associated with a 3.84% increase (95% bayesian credible interval [CrI], 1.83%-5.89%) in psychoactive drug–related death rates in the month of exposure, persisting up to 3 months after exposure (2.39% [CrI, 0.41%-4.40%]). Older children and younger adults (15-44 years old) demonstrated statistically higher increases than older adults after exposure. However, increases were limited to low-poverty counties regardless of racial composition (13.05% [CrI, 6.79%-19.68%] for low presence of racial and ethnic minority residents; 6.01% [CrI, 3.02%-9.08%] for high presence) in the month of tropical cyclone exposure.

**Conclusions and Relevance:**

In this case-control study of US counties, tropical cyclone exposure was associated with increased psychoactive drug–related death rates up to 3 months after exposure. These findings support the integration of substance use and mental health services into climate disaster preparedness and response planning.

## Key Points

**Question:**

In the US during 1988 to 2019, was county-level tropical cyclone exposure associated with increases in county-level psychoactive drug–related death rates in subsequent months?

**Findings:**

In this case-control study of 798 691 psychoactive drug–related deaths in 1258 counties that experienced tropical cyclones from 1988 to 2019, each additional cyclone-exposed day per month was associated with a 3.84% increase in death rates in the month of cyclone exposure and a 3.76% increase in the month after.

**Meaning:**

Among US counties exposed to tropical cyclones from 1988 to 2019, each additional cyclone-exposed day per month was associated with an increase in psychoactive drug–related death rates in the months following exposure.

## Teaser

This case-control study examines whether county-level tropical cyclone exposure is associated with changes in psychoactive drug–related death rates in the US.

## Introduction

Hurricanes and other tropical cyclones are among the most destructive weather events in the US,^1^ where half of the population lives in cyclone-exposed counties.^2^ Annually, tropical cyclones result in widespread service disruption, displacement, illness, and mortality.^3,4,5,6^ Mental and behavioral health consequences of tropical cyclones have been recorded, particularly after recent hurricanes.^7,8,9,10,11,12^ Most existing evidence comes from short-term case studies on posttraumatic stress,^7,10,13,14^ anxiety-mood disorders,^7,8,14^ and interpersonal violence.^15^ Psychoactive drug–related deaths represent a plausible downstream consequence of tropical cyclone exposure, yet they remain understudied.

Psychoactive drugs, including alcohol, certain prescribed medications, and some unregulated drugs,^16,17^ affect mood, cognition, and behavior and can be used to cope with psychological distress.^18,19^ Psychoactive drug–related death rates have risen sharply in the US since 2000,^20,21^ contributing to a public health crisis.^22^ Tropical cyclones, which have increased in strength,^23^ intensity,^24^ duration,^25^ and activity^26^ over recent decades, may exacerbate this crisis through 2 plausible pathways. These exposures can trigger acute psychological distress that may increase substance use and overdose risk,^27,28,29^ and they can disrupt access to health care and substance use treatment, which may be life-threatening for individuals with severe substance-related conditions.^30,31,32^ The objective of this study was to estimate the association between tropical cyclone exposure and psychoactive drug–related death rates in US counties over a 32-year period. The association was evaluated by age group, sex, and social disadvantage, which influence death rates^33,34^ and may modify tropical cyclone associations.

## Methods

This case-control study was approved by Columbia University’s institutional review board and was classified as non–human participant research; informed consent was therefore not required. This study followed the Strengthening the Reporting of Observational Studies in Epidemiology (STROBE) reporting guideline.

### Study Population

Complete death records from the National Center for Health Statistics (NCHS) were used to identify monthly cause-specific deaths by age, sex, race, and county of residence in the US during January 1, 1988, to December 31, 2019. Data were restricted to counties that experienced at least 1 tropical cyclone during the study period (1258 counties). County-level annual population data were obtained from the US Census Bureau for 1988 to 1989 and the NCHS bridged-race dataset for 1990 to 2019. Annual county-level population counts were assigned to June and linearly interpolated to calculate monthly population counts. County-level social disadvantage was evaluated via the percentage of residents in poverty (US Census Bureau 2018)^35^ and the percentage of racial and ethnic minority residents (ie, residents categorized as a group other than White) (Centers for Disease Control and Prevention 2018).^36^ Social disadvantage variables were dichotomized according to the 2018 median value among included counties.

### Exposure Assessment

Data on all tropical cyclones in the contiguous US during 1988 to 2019 were obtained, measured by maximum sustained wind speed at each county’s population mean center.^37,38,39^ Cyclone exposure was defined as the monthly number of days when a county experienced sustained winds of at least 34 knots.^38^ Associations were examined by cyclone strength (hurricanes: ≥64 knots, gale to violent storms: 34-63 knots). A national dataset with area-weighted monthly mean temperatures by county was obtained from the Parameter-elevation Regressions on Independent Slopes Model.^40^ Further details on all data and statistical analyses are included in the eAppendix in Supplement 1.

### Outcomes

NCHS mortality data include the underlying cause of death, which was classified according to the International Classification of Diseases (ICD) system (*International Classification of Diseases, Ninth Revision *[*ICD-9*] before 1999 and *International Statistical Classification of Diseases and Related Health Problems, Tenth Revision *[*ICD-10*] thereafter).^41,42^ Psychoactive drug–related deaths were defined to include poisoning by and exposure to noxious substances (ie, overdoses), mental and behavioral disorders due to psychoactive substance use, and alcohol-induced deaths.^41,42,43^ The corresponding ICD codes are described in eTable 1 in Supplement 1. All main analyses were conducted with total counts of psychoactive drug–related deaths.

### Statistical Analysis

Analyses were conducted between March 27, 2024, and June 25, 2025. To evaluate the county-level association between the number of tropical cyclone-exposed days and monthly psychoactive drug–related death rates, a bayesian formulation of the conditional quasi-Poisson model was applied, consistent with several previous studies.^4,5,6^ This model conditions on county-month strata, comparing each county-month containing cyclone-exposed days (cases) to its own baseline rate during the same calendar month in all other years without exposure (controls). The approach implements the matching inherent to a time-stratified case-crossover design, which adjusts for all time-invariant county characteristics as well as seasonality. The quasi-Poisson specification accommodates overdispersion in mortality counts, and the bayesian formulation allows for full distributional estimation of model parameters and borrowing of strength across neighboring units, helping to stabilize estimates in strata with sparse data.^5^

Time-varying confounding was addressed by adjusting for long-term trends and temperature. Psychoactive drug–related death rates have changed over time due to factors unrelated to tropical cyclone exposure, including consumption patterns, shifts in drug supply,^44^ and prescribing patterns.^45^ Temperature also influences both tropical cyclone formation and activity^46^ and the behavioral risks and physiological effects of drug use.^47^ To control for these factors, long-term trends were modeled using natural splines and temperature using a second-order random walk with weakly informative priors. To allow for distinct temporal patterns before and after the 2015 national rise in opioid mortality,^48^ a piecewise modeling approach was implemented with 2 separate splines for the 1988 to 2015 and 2016 to 2019 periods. A county-month-specific population offset was included to calculate death rates.^5^ Unconstrained distributed lag terms captured the association between each additional tropical cyclone–exposed day and drug-related death rates up to 3 months post cyclone,^5^ when acute disruptions in mental health, drug markets, substance use patterns, and health care access are most likely.^31,49^ This approach estimated 1 exposure-outcome association per month after tropical cyclone exposure while flexibly adjusting for nonlinear, period-specific trends.^50^

Analyses were conducted by tropical cyclone strength (all, gale to violent storms, and hurricanes), with further analyses stratified by age group (15-29, 30-44, 45-59, and ≥60 years), sex (female and male), and county-level social disadvantage. Social disadvantage was constructed by dichotomizing poverty and the presence of racial and ethnic minority residents according to the variable medians among included counties (above or below 16% in poverty; above or below 18% racial and ethnic minority residents) and categorizing combinations of both. This resulted in 4 categories: low poverty-low minority, low poverty-high minority, high poverty-low minority, and high poverty-high minority.

Results are presented as the mean percentage change in psychoactive drug–related death rates per additional tropical cyclone–exposed day in a month and the 3 months thereafter. Corresponding changes in monthly deaths per 1 000 000 population (DPM) were calculated by multiplying each posterior relative change by the median age-standardized monthly psychoactive drug–related death rate for 2019 (eTable 2 in Supplement 1). Associations were reported as positive when the 95% bayesian credible interval [CrI] was entirely nonnegative and negative when entirely nonpositive; CrIs spanning the null were interpreted as not statistically meaningful. Differences between subgroups were formally evaluated by estimating the posterior mean difference between group-specific log-rate ratios and their CrIs.

To assess the robustness of the estimated associations, several sensitivity analyses were conducted. First, the month structure was varied to test models with different post-cyclone exposure windows, and multiple time frames were compared to evaluate the effect of piecewise modeling decisions. Social disadvantage stratifications were based on county-level median values of poverty and percentage of racial and ethnic minority residents in 2018, the latest available year. To assess temporal stability, Pearson correlation coefficients were calculated between 1995 and 2018 poverty and racial and ethnic minority values. A leave-one-out analysis was conducted by omitting one state at a time from the main model to assess influence by individual states. Subcause sensitivity analysis was performed to compare the estimated association across death causes classified as more acute (eg, drug overdoses) vs more chronic (eg, alcoholic liver disease). An ICD-version sensitivity analysis was conducted by comparing results from the full study period with results limited to the *ICD-10*–only period to assess potential coding-related bias.

Statistical analyses were conducted via R Statistical Software, version 3.6.3 (R Foundation), integrated nested Laplace approximation in R-INLA, version 21.03.17. There were missing population data by race for Connecticut’s 8 counties (0.01% of observations); Connecticut was therefore excluded from social disadvantage-stratified analysis.

## Results

### Tropical Cyclones

**Figure 1.**
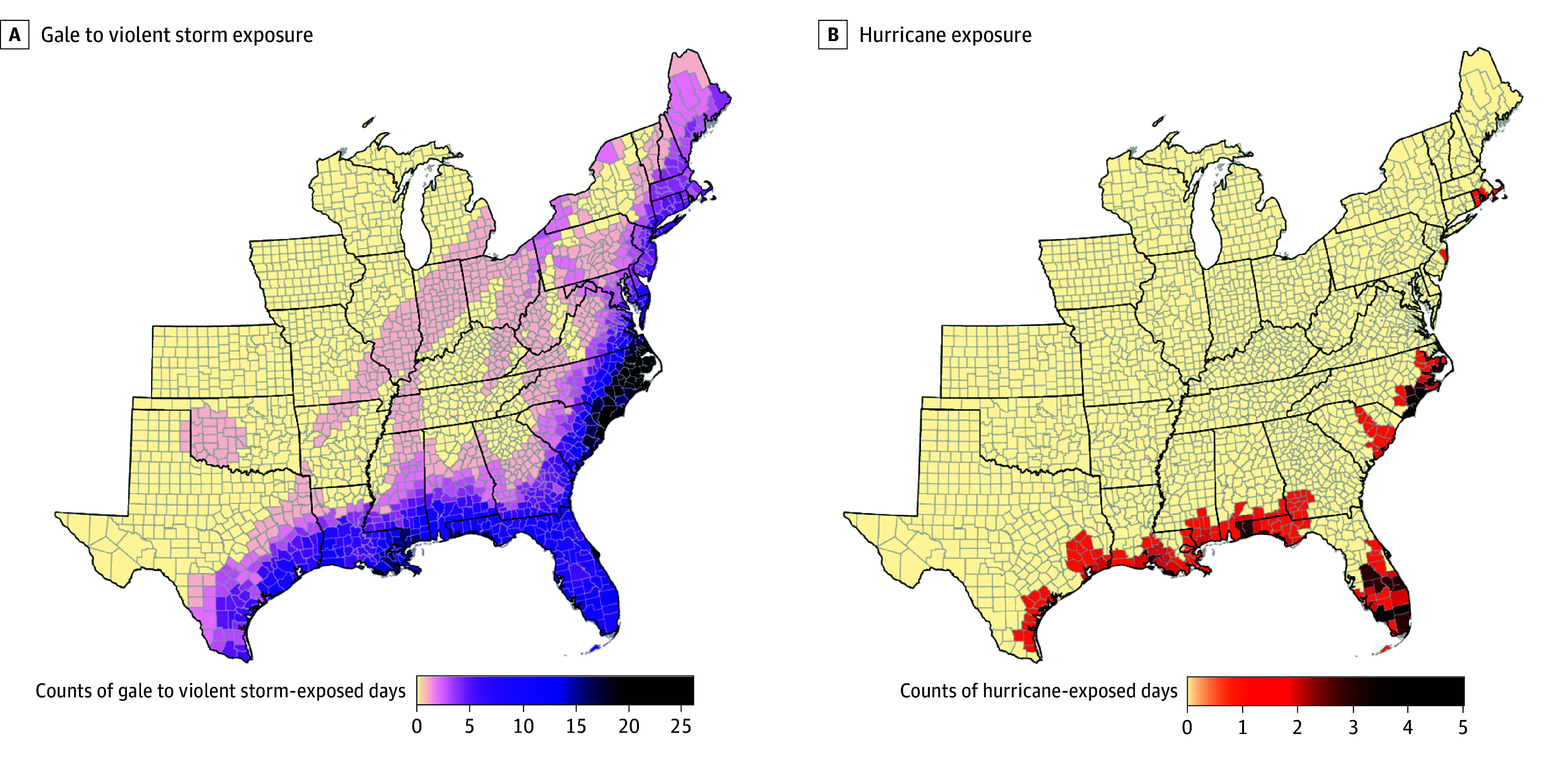
Tropical Cyclone Exposure Counts by US County, 1988-2019 A, Number of total gale to violent storm (34-63 knots) exposure days (n = 5072) and B, number of total hurricane (≥64 knots) exposure days (n = 233), depicted by county for the 1258 exposed counties during 1988 to 2019.

A total of 1258 counties were exposed to at least 1 cyclone during 1988 to 2019, representing 48.5% of the 2019 US population (Figure 1), with a total of 5305 tropical cyclone-exposed days in 5168 county-months. Exposure days consisted of 5072 gale to violent storm–exposed days in 4951 county-months across all included counties and 233 hurricane-exposed days in 229 county-months across 154 counties. Overall median exposure was 2 tropical cyclone days (range, 1-27 days). Gale to violent storm–exposed days ranged from 1 to 26, with a median of 2 (mean = 4.0). Gale to violent storms occurred in May through November, peaking in September (n = 2167 days). Hurricane-exposed days ranged from 1 to 5, with a median of 1 (mean = 1.5). Hurricanes occurred in July through October, also peaking in September (n = 98 days).

### Deaths

During 1988 to 2019, in tropical cyclone–exposed US counties, there were 798 691 total psychoactive drug–related deaths (eTable 3 in Supplement 1). There were 235 455 deaths (29.5%) among females and 563 236 (70.5%) among males. A total of 797 030 deaths (99.8%) were among those aged 15 years or older. Drug overdose was the leading cause (463 789 [58.1%]), followed by alcoholic liver disease (197 217 [24.7%]).

### Association of Tropical Cyclones With Psychoactive Drug–Related Death Rates

**Figure 2.**
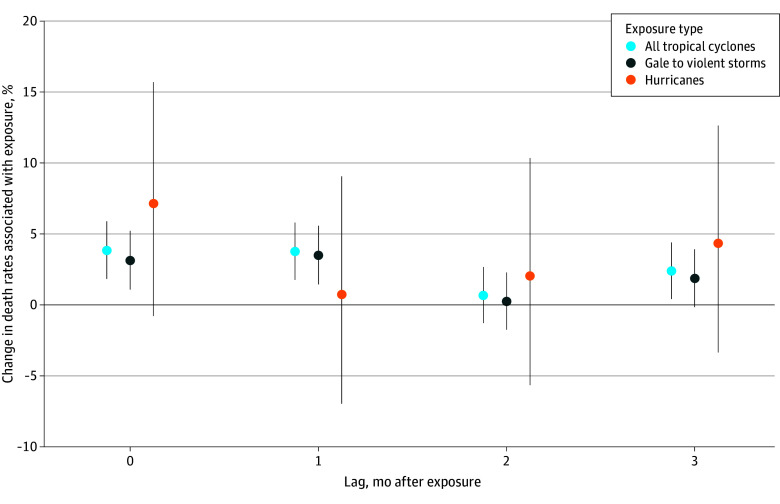
Percentage Change in Death Rates Per 1-Day Increase in Monthly Tropical Cyclone Exposure by Storm Category and Months After Exposure Dots indicate the point estimates, representing the mean; whiskers, 95% credible intervals.

Each additional day of tropical cyclone exposure was associated with a mean increase of 3.84% (CrI, 1.83%-5.89%) in psychoactive drug–related death rates in the month of cyclone exposure, which translates to an additional 5.37 DPM (Figure 2; eTable 4 in Supplement 1). The overall positive association persisted up to 3 months (2.39% [CrI, 0.41%-4.40%]) post cyclone. Gale to violent storms were associated with increases in the month of exposure (3.13% [CrI, 1.08%-5.22%]) and the month following exposure (3.49% [CrI, 1.45%-5.58%]). The highest point estimate was observed for hurricanes in the month of exposure (7.14% [CrI, −0.78% to 15.69%]), though the CrI included the null.

### Association of Tropical Cyclones With Psychoactive Drug–Related Death Rates by Age Group

**Figure 3.**
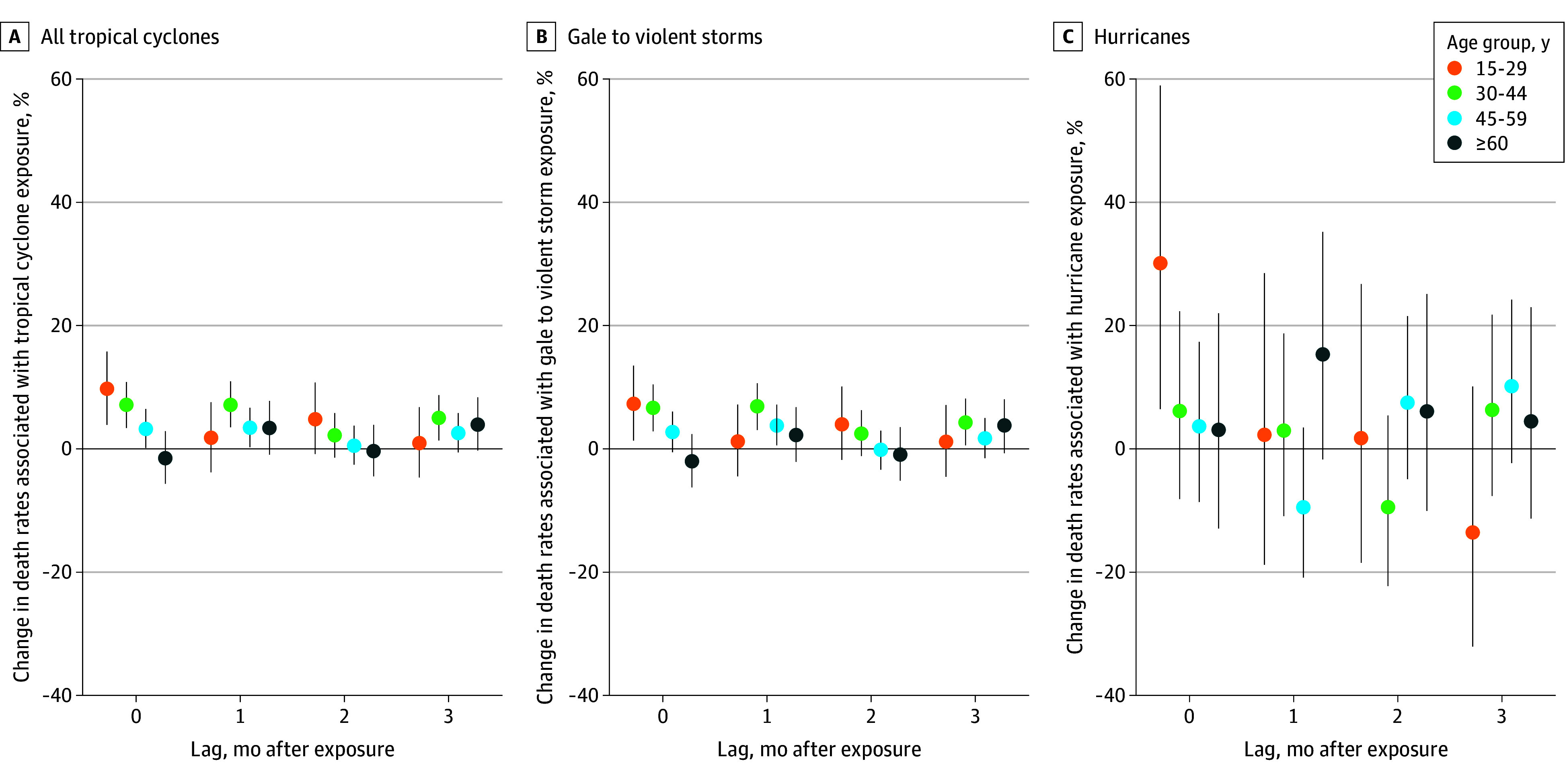
Percentage Change in Death Rates Per 1-Day Increase in Monthly Tropical Cyclone Exposure by Age Group, Storm Category, and Months After Exposure Dots indicate the point estimates, representing the mean; whiskers, 95% credible intervals.

Positive associations were observed across most age groups in the month of exposure for all cyclones and gale to violent storms, with the largest increases among those aged 15 to 29 years in the month of tropical cyclone exposure (9.67% [CrI, 3.84%-15.85%] and 11.72 [CrI, 4.64-19.19] additional DPM for all cyclones; 7.26% [CrI, 1.37%-13.50%] and 8.79 [CrI, 1.66-16.34] additional DPM for gale to violent storms) (Figure 3; eTable 5 in Supplement 1). Increases in death rates persisted at 1 month after all cyclones for those aged 30 to 44 years (7.16% [CrI, 3.49%-10.96%]) and 45 to 59 years (3.42% [CrI, 0.24%-6.71%]), with point estimates indicating the association attenuated with increasing age group. The only hurricane-force increase clear of the null was among those aged 15 to 29 years in the month of exposure (30.05% [CrI, 6.43%-58.92%]). For gale to violent storms and all tropical cyclones, the 15- to 29-year and 30- to 44-year age groups had larger posterior estimates than adults aged 60 years or older across all lags (eTable 6 in Supplement 1).

### Association of Tropical Cyclones With Psychoactive Drug–Related Death Rates by Sex

**Figure 4.**
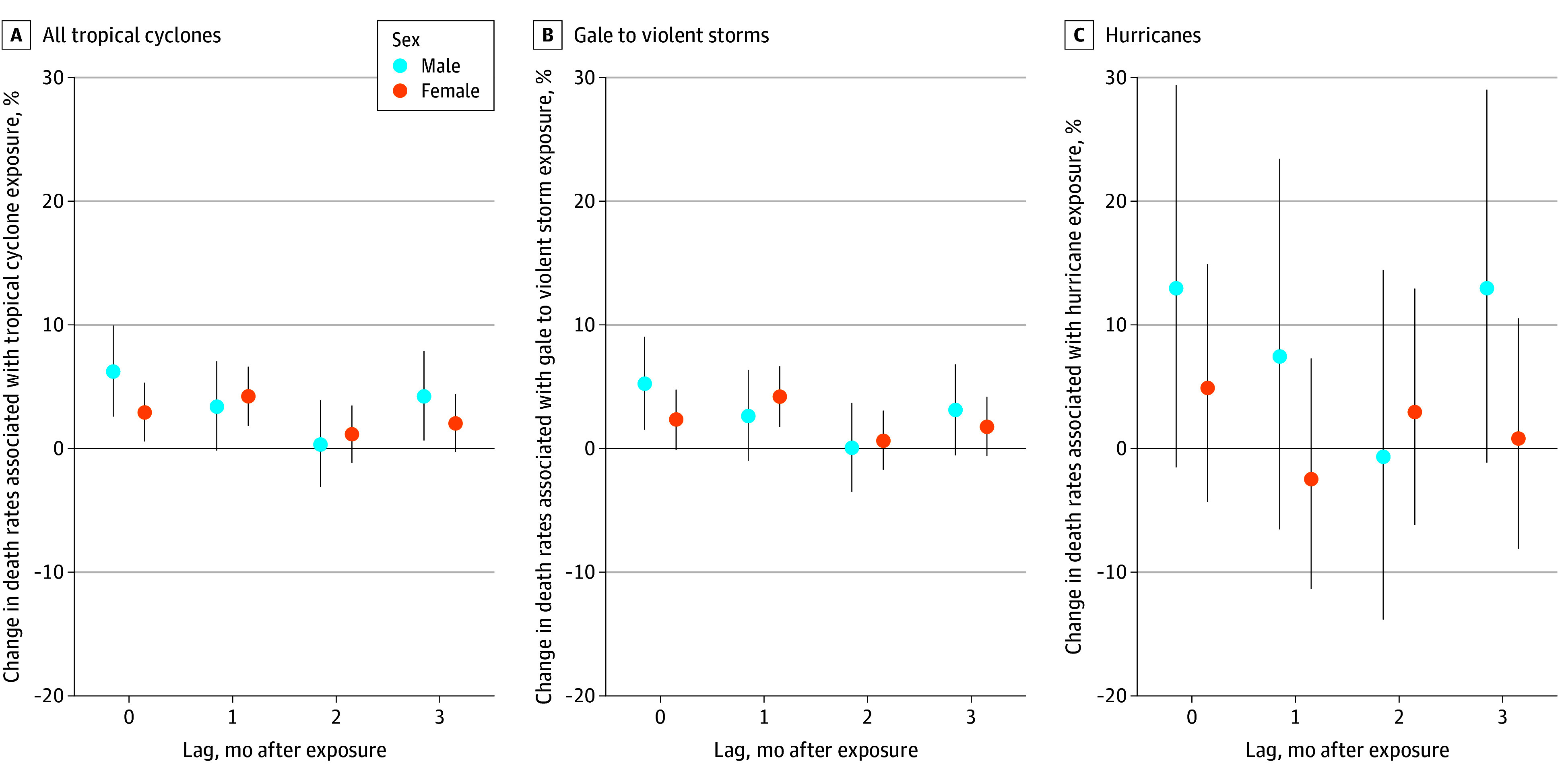
Percentage Change in Death Rates Per 1-Day Increase in Monthly Tropical Cyclone Exposure by Sex, Storm Category, and Months After Exposure Dots indicate the point estimates, representing the mean; whiskers, 95% credible intervals.

For all tropical cyclones, the estimated increase in deaths was 6.19% (CrI, 2.54%-9.97%) for females ((7.71 [CrI, 3.17-12.42] additional DPM) compared with 2.92% (CrI, 0.55%-5.34%) for males (4.29 [CrI, 0.81-7.86] additional DPM) in the month of exposure (Figure 4; eTable 7 in Supplement 1). A similar pattern was observed for gale to violent storms. For hurricanes, there were no discernible changes from the null for either sex (12.88% [CrI, −1.56% to 29.42%] for females; 4.85% [CrI, −4.37% to 14.96%] for males). Across all cyclone strengths and lags, there were no discernible differences between males and females.

### Association of Tropical Cyclones With Psychoactive Drug–Related Death Rates by Social Disadvantage

**Figure 5.**
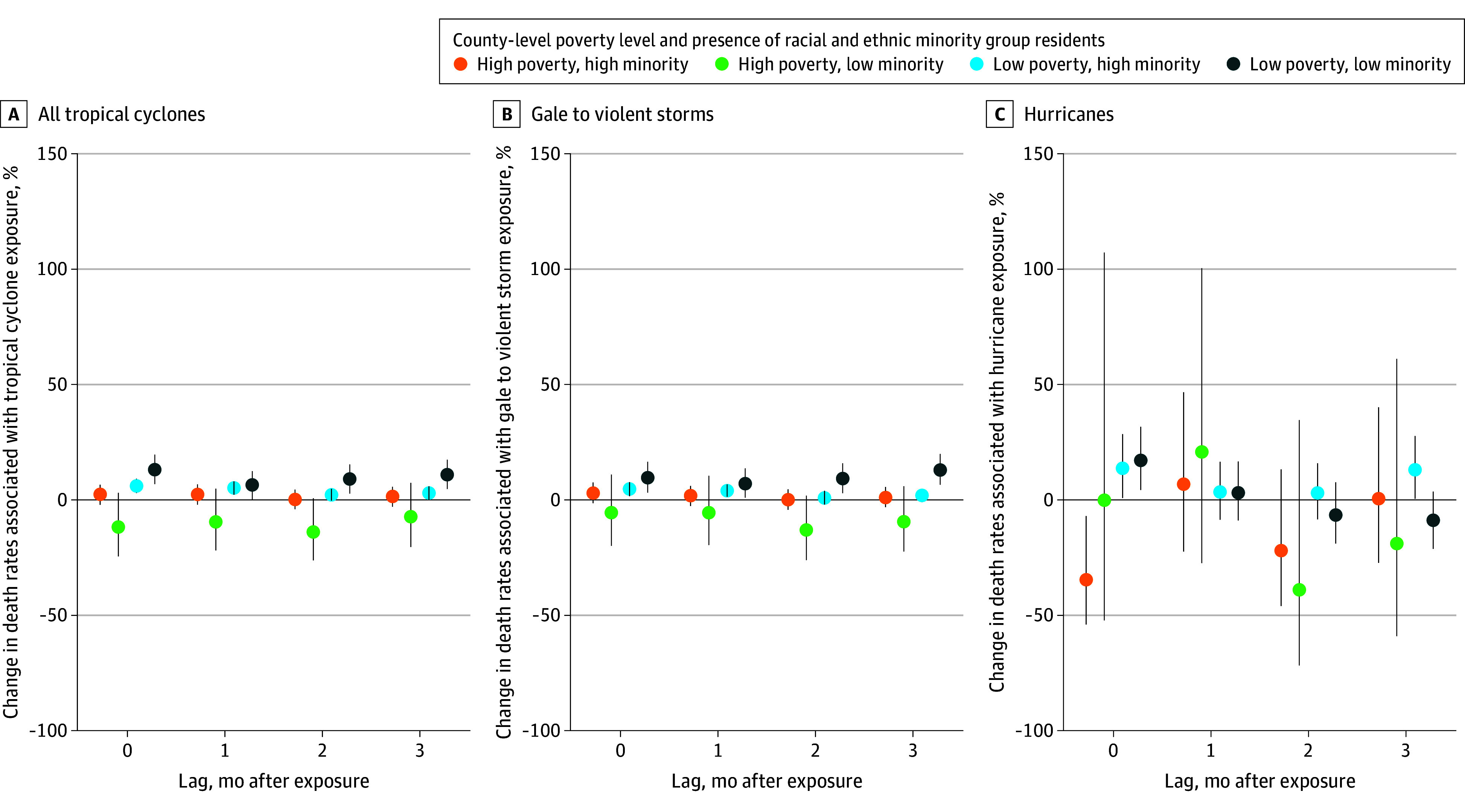
Percentage Change in Death Rates Per 1-Day Increase in Monthly Tropical Cyclone Exposure by Poverty, Presence of Racial and Ethnic Minority Residents, Storm Category, and Months After Exposure Poverty is defined as the 2018 county-level percentage of residents below the poverty threshold. Presence of racial and ethnic minority residents is defined as the 2018 county-level percentage of residents who are not racially categorized as White. Both variables were dichotomized according to the variable median (high = above median percentage, low = equal to or below median percentage). Dots indicate the point estimates, representing the mean; whiskers, 95% credible intervals.

Of the 1258 exposed counties, 400 (32%) were categorized as low poverty-low minority, 234 (19%) as low poverty-high minority, 225 (18%) as high poverty-low minority, and 391 (31%) as high poverty-high minority. In the month of exposure to all tropical cyclones, low poverty-low minority (mean increase, 13.05% [CrI, 6.79%-19.68%]; 22.82 [CrI, 11.87-34.41] additional DPM) and low poverty-high minority (mean increase, 6.01% [CrI, 3.02%-9.08%]; 7.08 [CrI, 3.56-10.70] additional DPM) counties experienced positive associations, while high poverty county estimates were null (Figure 5; eTable 8 in Supplement 1). Similar results were found in the month of gale to violent storm exposure, with low poverty-low minority counties having the greater positive association (9.58% [CrI, 3.23%-16.32%]), and high-poverty counties being null. In the month of hurricane exposure, estimates were 17.12% (CrI, 4.16%-31.70%) for low poverty-low minority, 13.68% (CrI, 0.49%-28.59%) for low poverty-high minority, and null or negative for high-poverty counties. Across lags, posterior mean differences demonstrated that low-poverty counties had greater increases in psychoactive drug–related death rates than high-poverty counties for all tropical cyclones and hurricanes (eTable 6 in Supplement 1).

### Sensitivity Analyses

Across alternative post-exposure windows, associations remained consistently positive and similar in magnitude (eFigure 1 in Supplement 1). For piecewise model specification, the selected division (1988-2015 and 2016-2019) was chosen to include the most years without introducing influence from the COVID-19 pandemic (eFigure 2 in Supplement 1). High correlations were observed between 1995 and 2018 county-level measures of poverty (*r* = 0.89) and percentage of racial and ethnic minority residents (*r* = 0.98) (eFigures 3 and 4 in Supplement 1), and a bivariate map illustrated their geographic codistribution (eFigure 5 in Supplement 1). Leave-one-out analyses showed minimal influence of individual states, except for Florida and New York (eFigure 6 in Supplement 1). Subcause analyses indicated positive associations for both acute causes and chronic conditions, supporting the grouping of these causes of death despite the differing mechanisms (eFigure 7 in Supplement 1). Findings were robust when restricted to the *ICD-10* period (eFigure 8 in Supplement 1). Overall, sensitivity analyses supported the main findings. A directed acyclic graph depicting the hypothesized exposure-outcome relationship is provided (eFigure 9 in Supplement 1).

## Discussion

Among cyclone-exposed US counties during 1988 to 2019, each additional tropical cyclone–exposed day per month was associated with increases in psychoactive drug–related death rates. The associations were greatest in the month of exposure and, in some cases, persisted for up to 3 months post cyclone.

Tropical cyclone exposure may plausibly influence psychoactive drug–related deaths.^5,51^ Tropical cyclones can generate environmental and socioeconomic disruptions at both the community level (eg, displacement, food insecurity)^29^ and the individual level (eg, traumatic experiences, job loss).^7,28,52^ These adverse environmental and socioeconomic impacts have been associated with psychological distress,^27,28,29^ anxiety,^28,53^ depression,^28,29^ and grief,^28,54^ all of which can lead to the use of alcohol^55^ and other psychoactive drugs^56^ as a coping behavior.^55^ Tropical cyclone exposure can also directly trigger psychological distress due to witnessing and/or experiencing such an extreme weather event.^13^ This experience of climate-related traumatic stress^57^ may likewise lead to increased psychoactive drug use.^18,19^

Acute psychological responses may occur immediately after exposure, whereas tropical cyclones may also affect longer-term, chronic^30,58,59^ substance-related disease processes^60^ by disrupting access to health care,^31,32,60^ harm reduction services,^51,60^ and substance use treatment.^60^ These disruptions can exacerbate underlying vulnerabilities, especially in advanced cases (eg, alcoholic cardiomyopathy), by limiting access to medications or medical care.^59^ Tropical cyclones may also destabilize local drug markets by limiting supply chains and shifting drug availability, potentially increasing the risk of exposure to more potent or adulterated substances (eg, fentanyl) during periods of scarcity.^29,61^ Together, these compounding disruptions may help explain the elevated risk of psychoactive drug–related deaths observed in the months following cyclone exposure.

Increases in psychoactive drug–related mortality were evident across most age groups, but posterior comparisons showed that older children and younger adults (15-44 years) exhibited larger increases than adults aged 60 years or older, indicating greater sensitivity to cyclone-related disruptions in these younger populations. Younger people more frequently use prescription or illicit drugs in response to acute stressors,^62^ which may heighten overdose risk immediately after cyclones. For those aged 30 to 59 years, elevated death rates persisted at 1 month post cyclone, potentially reflecting sustained psychological distress given employment instability and caregiving burdens^63,64^ and/or unmanaged chronic or advanced diseases given prolonged health care disruptions.^59,60^ The sex-specific analysis indicated no discernible differences, as all CrIs for posterior mean differences included the null. The larger relative increase among females likely reflects their lower baseline psychoactive drug–related death rates, meaning modest absolute increases can yield higher percent changes than in males.^65^

The largest increases in death rates occurred in low poverty-low minority counties, and posterior differences indicated that these increases were statistically greater than high-poverty counties for all tropical cyclones and hurricanes. These findings may reflect preexisting patterns of substance use and substance use disorders, access to drugs, and local drug market dynamics. Lower-poverty, predominantly White communities have greater access to prescription drugs,^66^ higher rates of prescription drug misuse concurrent with illicit drug use,^67^ and higher rates of substance use disorders,^68^ which may increase the risk of misuse or overdose when health care systems are disrupted and prescriptions become unmonitored. Additionally, individuals with higher socioeconomic status are more likely to use tranquilizers (eg, Xanax),^69^ stimulants (eg, cocaine),^69,70^ and alcohol.^70^ The ability to purchase and access these substances may be greater in wealthier communities, and disruptions in local drug markets during and after cyclones may increase reliance on more dangerous or adulterated substances.^29,61^ In contrast, high-poverty communities with larger proportions of racial and ethnic minority residents may face limited access to both formal mental health services^71^ and prescription drugs.^66^ These communities may rely more on informal support systems^72^ and may also have less purchasing power or fewer connections to illicit drug supply chains.^73^ This may reduce the likelihood of increased drug consumption, potentially explaining the observed decrease in psychoactive drug–related deaths in the month of a hurricane. Residents of high-poverty communities may also be more likely to die from other causes, such as cardiovascular disease or diabetes,^74^ due to chronic condition exacerbation given cyclone-related health care disruptions.^30,31,32,59,60^

### Strengths and Limitations

A strength of this study is its leveraging of 3 decades of national mortality data. This study is, to our knowledge, the first to quantify the association between tropical cyclone exposure and psychoactive drug–related deaths broadly and by key sociodemographic variables. This study also has several limitations. First, exposure misclassification is possible, as county-level residence may not reflect individuals’ physical location at the time of cyclone exposure. Second, the cyclone exposure metric was based solely on wind speed and did not account for other cyclone-related hazards such as flooding or infrastructure damage. Though we did not study tornadoes, a similar framework would be applicable. Third, despite model adjustment, there may be residual confounding by unmeasured variables, though they would need to covary with both cyclone exposure and drug-related death rates while remaining independent of included covariates, which is unlikely. Fourth, outcome misclassification is possible, particularly among older adults, whose drug-related deaths may be misattributed to preexisting conditions.^75^ Fifth, the geographic scope excluded Alaska, Hawaii, and US territories, which have experienced substantial recent cyclone exposures.^51^ Sixth, because analyses were conducted at the county level, county-level associations may not reflect individual-level relationships. Finally, the use of counties as the unit of analysis may not capture within-county variation, likely producing nondifferential exposure misclassification and attenuating estimates toward the null.

## Conclusions

In this case-control study of US counties, tropical cyclone exposure was associated with increased psychoactive drug–related death rates up to 3 months post exposure. These findings support the integration of substance use and mental health services into climate disaster preparedness and response planning.

## Notes

Supplement 1. **eAppendix.** Supplemental methods
eReferences
**eTable 1. **Causes of death used in the analysis with ICD-9 and ICD-10 codes **eTable 2. **Age-standardized psychoactive drug-related death rate by percentile of the tropical cyclone-exposed county units used in the analysis at the end of the study period (2019). **eTable 3. **Summary statistics for psychoactive drug-related deaths by subcause in the 1,258 United States counties included in this analysis, 1988-2019. **eTable 4. **Main analysis **eTable 5. **Age-stratified analysis **eTable 6**. Posterior mean differences in log-rate ratios across demographic and social disadvantage groups following tropical cyclone exposure **eTable 7. **Sex-stratified analysis **eTable 8. **Social disadvantage-stratified analysis **eFigure 1. **Sensitivity analysis for model lag specifications **eFigure 2. **Sensitivity analysis for time periods to be used in piecewise modeling **eFigure 3.** Comparison of median percentage of residents in poverty for 1995 and 2018 for included U.S. counties (n=1,258) **eFigure 4.** Comparison of median percentage of racial and ethnic minority residents for 1995 and 2018 for included U.S. counties with racial composition data (n=1,250) **eFigure 5. **Bivariate map of county-level social disadvantage (intersection of poverty and presence of racial and ethnic minority residents) and tropical cyclone exposure in U.S counties exposed to at least one tropical cyclone during 1988-2019, for all tropical cyclone exposures and for hurricane exposures only **eFigure 6.** Leave-one-out sensitivity analysis to assess individual U.S. state influence on the estimated national association (n=34) **eFigure 7.** Subcause sensitivity analysis to compare the estimated association across death causes classified as more acute (e.g., drug overdoses) versus more chronic (e.g., alcoholic liver disease) **eFigure 8.** Sensitivity analysis to assess whether the association changed significantly between ICD-versions 9 and 10, comparing 1988-2019 to 2000-2019 **eFigure 9.** Directed acyclic graph depicting the epidemiologic relationship between county-level tropical cyclone exposure and county-level psychoactive drug-related death rates

Supplement 2.
Data Sharing Statement
