# Supplementary material for: Tropical Cyclone Exposure and Psychoactive Drug–Related Death Rates
Source: JAMA Netw Open. 2026 Feb 20;9(2):e2560183. doi: 10.1001/jamanetworkopen.2025.60183 (PMC12924108; doi:10.1001/jamanetworkopen.2025.60183)
Supplement: Supplement 1. — eAppendix. Supplemental methods eReferences eTable 1. Causes of death used in the analysis with ICD-9 and ICD-10 codes eTable 2. Age-standardized psychoactive drug-related death rate by percentile of the tropical cyclone-exposed county units used in the analysis at the end of the study period (2019). eTable 3. Summary statistics for psychoactive drug-related deaths by subcause in the 1,258 United States counties included in this analysis, 1988-2019. eTable 4. Main analysis eTable 5. Age-stratified analysis eTable 6. Posterior mean differences in log-rate ratios across demographic and social disadvantage groups following tropical cyclone exposure eTable 7. Sex-stratified analysis eTable 8. Social disadvantage-stratified analysis eFigure 1. Sensitivity analysis for model lag specifications eFigure 2. Sensitivity analysis for time periods to be used in piecewise modeling eFigure 3. Comparison of median percentage of residents in poverty for 1995 and 2018 for included U.S. counties (n=1,258) eFigure 4. Comparison of median percentage of racial and ethnic minority residents for 1995 and 2018 for included U.S. counties with racial composition data (n=1,250) eFigure 5. Bivariate map of county-level social disadvantage (intersection of poverty and presence of racial and ethnic minority residents) and tropical cyclone exposure in U.S counties exposed to at least one tropical cyclone during 1988-2019, for all tropical cyclone exposures and for hurricane exposures only eFigure 6. Leave-one-out sensitivity analysis to assess individual U.S. state influence on the estimated national association (n=34) eFigure 7. Subcause sensitivity analysis to compare the estimated association across death causes classified as more acute (e.g., drug overdoses) versus more chronic (e.g., alcoholic liver disease) eFigure 8. Sensitivity analysis to assess whether the association changed significantly between ICD-versions 9 and 10, comparing 1988-2019 to 2000-2019 eFigure 9. Directed acyclic graph [file jamanetwopen-e2560183-s001.pdf]

Supplementary Online Content

Spriggs R, Lynch VD, Lu Y, et al. Tropical cyclone exposure and psychoactive drug-related death rates. *JAMA Netw Open*. 2026;9(2):e2560183. doi:10.1001/jamanetworkopen.2025.60183

eAppendix. Supplemental methods.

eReferences.

eTable 1. Causes of death used in the analysis with ICD-9 and ICD-10 codes.

eTable 2. Age-standardized psychoactive drug-related death rate by percentile of the tropical cyclone-exposed county units used in the analysis at the end of the study period (2019).

eTable 3. Summary statistics for psychoactive drug-related deaths by subcause in the 1,258 United States counties included in this analysis, 1988-2019.

eTable 4. Main analysis.

eTable 5. Age-stratified analysis.

eTable 6. Posterior mean differences in log-rate ratios across demographic and social disadvantage groups following tropical cyclone exposure.

eTable 7. Sex-stratified analysis.

eTable 8. Social disadvantage-stratified analysis.

eFigure 1. Sensitivity analysis for model lag specifications.

eFigure 2. Sensitivity analysis for time periods to be used in piecewise modeling.

eFigure 3. Comparison of median percentage of residents in poverty for 1995 and 2018 for included U.S. counties (n=1,258).

eFigure 4. Comparison of median percentage of racial and ethnic minority residents for 1995 and 2018 for included U.S. counties with racial composition data (n=1,250).

eFigure 5. Bivariate map of county-level social disadvantage (intersection of poverty and presence of racial and ethnic minority residents) and tropical cyclone exposure in U.S counties exposed to at least one tropical cyclone during 1988-2019, for all tropical cyclone exposures and for hurricane exposures only.

eFigure 6. Leave-one-out sensitivity analysis to assess individual U.S. state influence on the estimated national association (n=34).

© 2026 Spriggs R et al. JAMA Network Open.

eFigure 7. Subcause sensitivity analysis to compare the estimated association across death causes classified as more acute (e.g., drug overdoses) versus more chronic (e.g., alcoholic liver disease).

eFigure 8. Sensitivity analysis to assess whether the association changed significantly between ICD-versions 9 and 10, comparing 1988-2019 to 2000-2019.

eFigure 9. Directed acyclic graph depicting the epidemiologic relationship between county-level tropical cyclone exposure and county-level psychoactive drug-related death rates.

This supplemental material has been provided by the authors to give readers additional information about their work.

© 2026 Spriggs R et al. JAMA Network Open.

eAppendix. Supplemental methods

eMethods 1. Details on tropical cyclone data, mortality data, covariate data, and statistical analysis

Data sources

Exposure

Data were obtained on tropical cyclone wind exposure in the contiguous United States, with full space and time coverage during 1988-2019, via the *hurricaneexposure* and *hurricaneexposuredata* R packages.[1,2] An exhaustive assessment of tropical cyclones was generated from those recorded in the HURDAT2 dataset based on wind field modeling and validation against observations from weather stations.[3] First, all tropical cyclones that came within 250 km of a US county were retained for further wind modeling.[1] For these, the wind field at each county's population mean center was modeled every 15 min, while the storm was tracked, providing an estimate of peak local winds that the storm brought to that county.[4] This modeling used a double exponential wind model to estimate 1-min surface wind at each county center, based on the storm's forward speed, direction, and maximum wind speed.[4,5] This tropical cyclone wind exposure dataset covers the 1,258 counties in 34 state or district units (33 states and Washington DC) with at least one tropical cyclone wind exposure. Daily estimates of maximum wind sustained speed by county were used to generate classifications of these exposures using cut points from the Beaufort wind scale.[6] The Beaufort scale is an empirical measure that relates locally measured wind speed to observed conditions on sea or land from calm to hurricane force. This analysis focused on tropical cyclone winds, i.e., ≥34 knots, which include both hurricanes and gale to violent storms. As with previous work,[7–9] hurricane exposure was defined as all days when the peak sustained wind in the county's population center reached or exceeded 64 knots (74 mph; hurricane-force wind on the Beaufort scale).[6] Similarly, gale to violent storm exposure was defined as all days which reached or exceeded 34 knots (39 mph; gale-force wind on the Beaufort scale),[6] yet did not reach 64 knots.

Temperature data was obtained from the Parameter-elevation Regressions on Independent Slopes Model (PRISM), which collects climate observations from a range of monitoring networks.[10] The model uses quality control measures to generate a national temperature dataset with full space and time coverage during the study period. Gridded daily estimates at 4-kilometer resolution were used to generate area-weighted monthly average temperatures by county.

Outcome

In counties that experienced at least one tropical cyclone during 1988-2019, data on psychoactive drug-related deaths from three major causes (drug overdoses, mental/behavioral disorders due to substance use, alcohol-induced deaths) by sex, age, underlying cause of death, and county of residence were obtained through the National Center for Health Statistics (NCHS) (https://www.cdc.gov/nchs/nvss/dvs_data_release.htm).

© 2026 Spriggs R et al. JAMA Network Open.

Population data were retrieved from the NCHS Vintage 2020 bridged-race dataset for 1990 to 2018 (https://www.cdc.gov/nchs/nvss/bridged_race.htm) and from the US Census Bureau prior to 1990 (https://www.census.gov/data/tables/time-series/demo/popest/1980s-county.html). Race bridging refers to making data collected using one set of race categories consistent with data collected using a different set of race categories, to allow for estimation.

The underlying cause of death was coded according to the International Classification of Diseases (ICD) system (9th revision from 1988 to 1998 and 10th revision thereafter) and World Health Organization Global Health Estimate cause categories (eTable 2).[11] Monthly population counts were calculated through linear interpolation, by assigning each yearly count to June.

Covariate data

The social disadvantage variables of interest include percentage in poverty and percentage of racial and ethnic minority residents. County-level data on the percentage of residents below the poverty threshold were obtained from U.S. Census Bureau, covering 1995-2018.[12] Similarly, county-level data on the percentage of racial and ethnic minority residents were obtained from the Centers for Disease Control and Prevention Wide-ranging Online Data for Epidemiologic Research (CDC WONDER), also covering 1995-2018.[13] Data from 2018 were used, the most recent year of available data, to conduct analysis stratified by social disadvantage.

Statistical analysis

The percent change in death rates associated with tropical cyclone exposure is modeled as:

$$\log(E[deaths_{ct}]) =$$

$$\alpha_0 + \alpha_{c\,m(t)} + \sum_{l=0}^{3} \beta_l Exposure_{lct} + f_{time}(t) + \tau_{temp_{ct}} + \log(Population_{ct})$$

where $deaths_{ct}$ is the number of cause-specific deaths in county *c* and month-year time *t* (ranging from 1 to 384, the total number of months in the study period); α_0 is the overall intercept and $\alpha_{c\,m(t)}$ is the county-month specific intercept, which is not estimated in the conditional Poisson model, where m(t) is a function that extracts the calendar month for time *t*; β_l is the lag-specific coefficient (log rate ratio) for tropical cyclone exposure in lagged month *l*; $Exposure_{lct}$ is the count of tropical cyclones in lagged month *l*, county *c,* at time *t*; $f_{time}(t)$ was modeled using two separate second-order random walk (RW2) components: one for 1988–2015 and one for 2016–2019. RW2 priors allow smooth nonlinear temporal adjustment within each period, capturing both gradual secular trends and medium-scale fluctuations. This specification provides stronger protection against confounding by the opioid and fentanyl epidemics and accommodates potential temporal autocorrelation. $\tau_{temp_{ct}}$ is a second-order random walk term (equivalent to a spline with equally-spaced knots[14]) to flexibly model the temperature-mortality relationship where $temp_{ct}$ is

© 2026 Spriggs R et al. JAMA Network Open.

the area-weighted monthly average temperature in county *c* and time *t*; and $Population_{ct}$ is the population in county c and time t, included as a population offset.

To further control for time-invariant county characteristics, we included a stratum term, defined as a unique indicator for each county-month combination. This term is equivalent to conditioning on county-month-specific intercepts in the conditional Poisson model. By construction, the stratum absorbs all stable or quasi-stable county characteristics (e.g., geographic position, coastal proximity, long-standing socioeconomic and demographic profiles, healthcare infrastructure, and persistent patterns of drug use) so that each county serves as its own control. This eliminates confounding by any factor that does not meaningfully vary within a county across months.

Operationally, the conditional Poisson likelihood conditions out a separate intercept for every county-month combination. Because a county-month intercept absorbs all factors that are constant within that stratum, the model estimates the tropical cyclone-mortality association by comparing exposed months in each county to unexposed months in the same county and calendar month across different years. This is mathematically equivalent to the matching performed in a time-stratified case-crossover design.

As in previous related work,[15,16] weakly informative priors were used for parameter estimation. For the RW2 components and county-month intercepts α_c and the second-order random walk term τ, hyper-priors were defined on the logarithm of the precision ($1/\sigma^2$) and modeled as *logGamma*(θ, δ) distributions with shape $\theta = 1$ and rate $\delta = 0.001$. All of the other terms, including the β_l parameters, were modeled with Gaussian priors N(0, 1000). Inference was conducted using the Integrated Nested Laplace Approximation (INLA), which provides accurate approximations to posterior marginal distributions.

The association was investigated for all tropical cyclone exposures together (≥34 knots), and stratified modeling was employed to separately estimate associations between exposure to hurricanes (≥64 knots) and gale to violent storms (34-63 knots) and psychoactive drug-related deaths (Figure 2 in main paper). Using stratified modeling, the association was evaluated for effect modification by age group (15-29 years, 30-44 years, 45-59 years, 60+ years; Figure 3 in main paper) and sex (female, male; Figure 4 in main paper).

The association was also assessed for effect modification by county-level social disadvantage. Social disadvantage was constructed by dichotomizing county-level percentage of residents below the poverty threshold and county-level percentage of racial and ethnic minority residents according to the 2018 variable medians. Among the 1,258 counties included in the main analysis, variable medians were 16% for poverty and 18% for racial and ethnic minority residents. Thus, counties with percentages above a variable median were categorized as high for each respective variable and counties with percentages equal to or below a variable median were categorized as low for each respective variable. Combinations of both low and high poverty and racial and ethnic minority residents were then categorized. This produced four categories: low poverty-low minority, low

© 2026 Spriggs R et al. JAMA Network Open.

poverty-high minority, high poverty-low minority, and high poverty-high minority. For each social disadvantage-stratum, the same model as described above with county-specific population offsets was applied (Figure 5 in main paper).

The reported 95% Bayesian credible intervals (CrI) are the 2.5th to 97.5th percentiles of each estimated parameter's posterior marginal distribution. Any reported positive association was based on a positive point estimate with a two-sided 95% credible intervals which were entirely non-negative (i.e., ranged from 0 to an upper bound), with a negative association the same but with a negative point estimate and a completely non-positive credible interval.

To formally evaluate whether tropical cyclone-mortality associations differed between subgroups (e.g., age, sex, or social disadvantage strata), posterior mean differences were calculated between pairs of subgroup-specific log-rate ratios. For each stratum, the Bayesian model produced a full posterior marginal distribution for the log-rate ratio at each lag. Posterior samples from two strata were then differenced pointwise (e.g., logRR_A -logRR_B), yielding a posterior distribution for the between-group contrast that incorporates uncertainty from both groups. The posterior mean of this difference and its 95% CrI were used to determine whether subgroup associations differed statistically. A subgroup difference was considered significant when the 95% CrI of the posterior mean difference did not include the null value (zero). This approach enables direct probabilistic comparison of tropical cyclone-related mortality effects across demographic and social disadvantage categories. These results are found in eTable 6.

eMethods 2. Statistical methods for the sensitivity analyses

Sensitivity analyses

For the lag structure sensitivity analysis, the Bayesian conditional quasi-Poisson model was re-estimated under varying specifications, ranging from a single-month lag when the exposure occurred (lag 0 only) to distributed lag structures extending up to six months post-exposure (lags 0–6). Each model maintained the same matching structure and covariate adjustments as the primary analysis. Posterior distributions were compared to evaluate consistency in the direction and magnitude of associations across lag choices, with particular attention to potential attenuation or inflation of estimates due to overlapping cyclone exposures across months. The estimates remained consistent across all lag specifications, indicating no confounding by correlated cyclone exposures (eFigure 1).

For the time period sensitivity analysis, separate models were estimated for four alternative temporal windows: 1988–2015, 1988–2021, 2016–2019, and 2016–2021. These models used similar model structure and exposure definitions as the main analysis, except there was no piecewise approach so long-term trends were modeled with a natural spline covering the entire time period under evaluation. Comparisons were made to determine whether the inclusion of post-

© 2026 Spriggs R et al. JAMA Network Open.

2019 data, which coincides with the onset of the COVID-19 pandemic, produced inflated or unstable estimates, potentially confounding the association between tropical cyclone exposure and psychoactive drug-related death rates. Models including 2020–2021 data showed increases in point estimates (eFigure 2), consistent with known COVID-era disruptions in mental health outcomes,[17] substance use patterns,[18] and population mobility.[19] Thus, years post-2019 were excluded from the final analysis to avoid time-varying confounding.

To assess the temporal stability of county-level poverty measures, the percentage of residents in poverty in 1995 was compared to that in 2018 for all included counties (n=1,258). A Pearson correlation coefficient was calculated, yielding a strong positive correlation (r = 0.89), indicating relative stability in poverty rankings over time (eFigure 3). Similarly, the percentage of county-level racial and ethnic minority residents in 1995 were compared to that of 2018 for all included counties with racial composition data, which excluded Connecticut's 8 counties (n=1,250). Correlation between the two time points was high (r = 0.98), suggesting minimal reordering of counties by racial composition (eFigure 4). These findings validated the use of 2018 variable medians as reasonable proxies to stratify counties by poverty and minority percentages throughout the 32-year study period.

We developed a bivariate map of social disadvantage (combination of poverty and minority residents) and tropical cyclone exposure to view the geographic distribution of these variables (eFigure 5). The map shows substantial geographic heterogeneity in the joint distribution of county-level poverty, racial composition, and tropical cyclone exposure among U.S. counties exposed at least once during 1988–2019. Along the Atlantic and Gulf coasts (where cyclone activity is most frequent) each of the four social disadvantage categories is represented. High-poverty–high-minority counties are concentrated in coastal Alabama, Louisiana, Georgia, South Carolina, and North Carolina, while high-poverty–low-minority counties appear along parts of Florida, the Carolinas, Virginia, Maryland, Delaware, and New Jersey. Low-poverty–low-minority counties are clustered primarily in Florida and Texas, and low-poverty–high-minority counties are visible in Texas, Florida, and parts of Maine. Although all SES groups experience cyclone exposure, the combination of Florida's large population, high frequency of cyclone and hurricane exposures, and substantial representation of low-poverty–low-minority counties in this high-exposure region likely contributes disproportionately to the observed SES-stratified associations. This spatial context helps clarify why the strongest increases in psychoactive drug-related mortality were detected in low-poverty strata despite coastal representation across all SES categories.

To evaluate whether any single state disproportionately influenced the national estimate, a leave-one-out sensitivity analysis was conducted, excluding one state at a time (n=34). Results were generally robust, with minimal variation in effect estimates (eFigure 6). Florida and New York exerted the greatest influence: omitting Florida reduced the magnitude of the national association in lag months 0 and 3, while omitting New York changed the direction of the estimate in lag month

© 2026 Spriggs R et al. JAMA Network Open.

2. The Florida results are consistent with our SES findings above. However, all leave-one-out estimates remained within the national model's 95% Bayesian credible interval, suggesting no single state unduly drove the findings.

To examine potential differences in the association by type of psychoactive drug-related death, analyses were repeated separately for a subset of acute (e.g., drug overdoses) and chronic (e.g., alcoholic liver disease) subcauses of death. Positive associations of varying magnitudes were observed for both acute and chronic causes (eFigure 7). This finding suggests that tropical cyclone exposure may contribute to psychoactive drug-related mortality through multiple pathways, including acute psychological distress and disruptions in care for chronic and advanced-stage substance-related conditions.

To test whether changes in diagnostic coding over time influenced the results, the main model was re-estimated using two time frames: 1988–2019 (including ICD-9 and ICD-10 periods) and 2000–2019 (ICD-10 only). Effect estimates were similar across both periods, and the direction and significance of the association remained unchanged (eFigure 8). This finding indicates that the observed associations were not materially affected by the transition from ICD-9 to ICD-10 coding.

Because psychoactive drug-related mortality exhibits strong long-term trends—including shifts associated with the opioid and fentanyl epidemics—we evaluated whether unmodeled temporal structure could bias the estimated associations as a sensitivity analysis. The temporal adjustment was strengthened by replacing the earlier fixed-effect spline terms with separate second-order random walk (RW2) components for the 1988–2015 and 2016–2019 periods. These RW2 terms provide flexible nonlinear adjustment for unmeasured temporal dependence within each period, allowing the model to account for gradual secular changes as well as shorter-term fluctuations that could induce autocorrelation in the residuals. The fitted standard deviations for the temporal RW2 components (0.0086 for 1988–2015; 0.0170 for 2016–2019) indicate modest temporal variability and appropriate smoothing of underlying patterns. Associations estimated under this enhanced specification were consistent with those obtained under the original spline-based model, demonstrating that the results were not driven by residual autocorrelation or coincident secular increases in drug-related mortality. The revised RW2 formulation therefore provides a more robust treatment of temporal confounding while preserving the substantive conclusions.

© 2026 Spriggs R et al. JAMA Network Open.

eReferences

1. Anderson GB, Ferreri J, Al-Hamdan M, et al. Assessing United States county-level exposure for research on tropical cyclones and human health. *Environ Health Perspect*. 2020;128(10):107009.

2. Anderson GB, Eddelbuettel D. Hosting data packages via drat: a case study with hurricane exposure data. *R J*. 2017;9(1):486.

3. Landsea C, Franklin J, Beven J. The revised Atlantic hurricane database (HURDAT2). Published online 2014. https://www.nhc.noaa.gov/data/hurdat/hurdat2-format-atlantic.pdf

4. Willoughby HE, Darling RWR, Rahn ME. Parametric Representation of the Primary Hurricane Vortex. Part II: A New Family of Sectionally Continuous Profiles. *Mon Weather Rev*. 2006;134(4):1102-1120. doi:10.1175/MWR3106.1

5. Anderson GB, Schumacher A, Guikema S, Quiring S, Ferreri J. Stormwindmodel: model tropical cyclone wind speeds. Published online 2020. https://cran.r-project.org/web/packages/stormwindmodel/index.html

6. National Meteorological Library and Archive. *National Meteorological Library and Archive Fact Sheet 6 — The Beaufort Scale*. MET Office; 2023. https://www.metoffice.gov.uk/binaries/content/assets/metofficegovuk/pdf/research/library-and-archive/library/publications/factsheets/factsheet_6-the-beaufort-scale_2023.pdf

7. Parks RM, Benavides J, Anderson GB, et al. Association of tropical cyclones with county-level mortality in the US. *JAMA*. 2022;327(10):946-955.

8. Parks RM, Anderson GB, Nethery RC, Navas-Acien A, Dominici F, Kioumourtzoglou MA. Tropical cyclone exposure is associated with increased hospitalization rates in older adults. *Nat Commun*. 2021;12(1):1545.

9. Parks RM, Kontis V, Anderson GB, et al. Short-term excess mortality following tropical cyclones in the United States. *Sci Adv*. 2023;9(33):eadg6633.

10. Daly C, Smith JI, Olson KV. Mapping Atmospheric Moisture Climatologies across the Conterminous United States. *PLoS One*. 2015;10(10):e0141140. doi:10.1371/journal.pone.0141140

11. Boerma T, Mathers CD. The World Health Organization and global health estimates: improving collaboration and capacity. *BMC Med*. 2015;13(1):1-4.

12. United States Census Bureau. Small Area Income and Poverty Estimates (SAIPE) Program. https://www.census.gov/programs-surveys/saipe/data/datasets.html

13. CDC. *CDC WONDER Online Databases*. Centers for Disease Control and Prevention; 2023. https://wonder.cdc.gov/DataSets.html

© 2026 Spriggs R et al. JAMA Network Open.

14. Rue H, Held L. *Gaussian Markov Random Fields: Theory and Applications*. CRC press; 2005.

15. Parks RM, Benavides J, Anderson GB, et al. Association of tropical cyclones with county-level mortality in the US. *JAMA*. 2022;327(10):946-955.

16. Kontis V, Bennett JE, Rashid T, et al. Magnitude, demographics and dynamics of the effect of the first wave of the COVID-19 pandemic on all-cause mortality in 21 industrialized countries. *Nat Med*. Published online October 14, 2020:1-10. doi:10.1038/s41591-020-1112-0

17. Chen PJ, Pusica Y, Sohaei D, Prassas I, Diamandis EP. An overview of mental health during the COVID-19 pandemic. *Diagnosis*. 2021;8(4):403-412.

18. Friedman J, Akre S. COVID-19 and the drug overdose crisis: uncovering the deadliest months in the United States, January–July 2020. *Am J Public Health*. 2021;111(7):1284-1291.

19. Berry CR, Fowler A, Glazer T, Handel-Meyer S, MacMillen A. Evaluating the effects of shelter-in-place policies during the COVID-19 pandemic. *Proc Natl Acad Sci*. 2021;118(15):e2019706118.

20. Keyes KM, Rutherford C, Smith GS. Alcohol-Induced Death in the USA from 1999 to 2020: a Comparison of Age–Period–Cohort Methods. *Curr Epidemiol Rep*. 2022;9(3):161-174.

© 2026 Spriggs R et al. JAMA Network Open.

eTable 1. International Classification of Diseases (ICD) underlying cause of death and subcause of death codes for psychoactive drug-related deaths. Included codes are referenced from the 9th revision, ICD-9, for 1988-1998 and 10th revision, ICD-10, for 1999-2019.

**Underlying cause of death**	**Subcause of death**	**ICD-9 codes**	**ICD-10 codes**
Alcohol-induced deaths[20]	Accidental poisoning by alcohol	E860	T51
Alcohol-induced deaths[20]	Alcohol-induced acute pancreatitis	N/A	K85.2
Alcohol-induced deaths[20]	Alcohol-induced chronic pancreatitis	N/A	K86.0
Mental/behavioral disorders due to substance use	Alcohol-induced mental/behavioral disorder	291, 303	F10
Alcohol-induced deaths[20]	Alcoholic cardiomyopathy	425.5	I42.6
Alcohol-induced deaths[20]	Alcoholic gastritis	535.5	K29.2
Alcohol-induced deaths[20]	Alcoholic liver disease	571.0-571.3	K70
Alcohol-induced deaths[20]	Alcoholic myopathy	N/A	G72.1
Alcohol-induced deaths[20]	Alcoholic polyneuropathy	357.5	G62.1
Alcohol-induced deaths[20]	Degeneration of nervous system due to alcohol	N/A	G31.2

© 2026 Spriggs R et al. JAMA Network Open.

Drug overdoses	Drug overdose	E850-E858, E980	X40-X49, Y10-Y19
Alcohol-induced deaths[20]	Excessive blood level of alcohol	790.3	R78.0
Mental/behavioral disorders due to substance use	Other psychoactive drug-induced mental/behavioral disorder	292, 304-305	F11-F19

© 2026 Spriggs R et al. JAMA Network Open.

eTable 2. Age-standardized psychoactive drug-related death rate by percentile of the tropical cyclone-exposed county units at the end of the study period (2019). To ensure stable rate estimates, age-standardized rates were calculated only for county × age × sex strata with populations ≥5,000, as very small strata produced unstable and inflated monthly rates when even a single death occurred.

Percentile		1st	5th	25th	50th	75th	95th	99th
Psychoactive drug-related monthly death rate (age standardized, per 1 million)	Overall	29.30	42.44	96.94	139.90	182.80	229.60	268.05
	Female	19.30	32.53	76.41	124.54	177.04	208.85	237.10
	Male	41.58	55.80	112.16	147.07	191.15	240.64	283.35
	0-14 years	12.70	14.93	26.66	55.57	90.43	149.15	155.00
	15-29 years	20.20	35.18	76.58	121.07	173.65	218.17	293.28
	30-44 years	30.04	45.77	95.22	136.38	178.07	241.58	327.32
	45-59 years	36.71	49.51	98.24	137.67	187.29	236.86	297.85
	60+ years	37.99	53.32	95.54	133.79	178.41	217.31	246.46
	High poverty - High minority	19.43	42.23	98.27	143.46	177.85	225.70	294.58
	High poverty - Low minority	48.47	57.05	112.06	161.46	210.38	272.32	313.50
	Low poverty - High minority	29.19	33.95	75.85	117.85	162.09	213.52	263.55
	Low poverty - Low minority	64.63	82.81	131.52	174.84	194.39	237.75	266.48

© 2026 Spriggs R et al. JAMA Network Open.

eTable 3. Summary statistics for psychoactive drug-related deaths by subcause in the 1,258 United States counties included in this analysis, 1988-2019.

Subcause of death	All deaths	Males	Females	0-14 years	15-29 years	30-44 years	45-59 years	60+ years
Accidental poisoning by alcohol	1,883	1,510	373	7	193	750	613	320
Alcohol-induced acute pancreatitis	1,890	1,471	419	0	91	471	869	459
Alcohol-induced chronic pancreatitis	1,497	1,158	339	1	41	347	705	403
Alcohol-induced mental/behavioral disorder	73,531	57,531	16,000	5	1,106	11,577	34,347	26,496
Alcoholic cardiomyopathy	9,726	8,453	1,273	0	100	1,489	3,853	4,284
Alcoholic gastritis	1,698	820	878	4	7	71	213	1,403
Alcoholic liver disease	197,217	143,460	53,757	4	1,377	31,707	91,345	72,784
Alcoholic myopathy	22	15	7	0	0	1	11	10
Alcoholic polyneuropathy	66	50	16	0	1	2	26	37
Degeneration of nervous system due to alcohol	1,220	1,008	212	0	4	57	319	840

© 2026 Spriggs R et al. JAMA Network Open.

Drug overdose	463,789	316,112	147,677	1,602	89,872	183,506	149,614	39,195
Excessive blood level of alcohol	50	39	11	1	9	18	14	8
Other psychoactive drug-induced mental/behavioral disorder	46,102	31,609	14,493	37	5,480	16,847	15,126	8,612
**Total deaths (Percentage)**	**798,691 (100%)**	**563,236 (70.5%)**	**235,455 (29.5%)**	**1,661 (0.0%)**	**98,291 (12.3%)**	**246,843 (30.9%)**	**297,055 (37.2%)**	**154,851 (19.4%)**

© 2026 Spriggs R et al. JAMA Network Open.

eTable 4. Percentage change in psychoactive drug-related death rates per one day increase in monthly tropical cyclone exposure by cyclone strength and lag time. Lag time was measured in months after tropical cyclone exposure. For each estimate, the corresponding change in monthly deaths per 1,000,000 population (DPM) and its 95% Bayesian credible interval are also reported. This Table accompanies Figure 2 from the main manuscript.

Cyclone strength	Lag (months)	Percentage change	95% Bayesian Credible Interval [CrI]		Additional DPM	DPM 95% Bayesian CrI	
All cyclones	0	3.84%	1.83%	5.89%	5.37	2.56	8.24
	1	3.76%	1.76%	5.80%	5.26	2.46	8.11
	2	0.67%	-1.28%	2.66%	0.94	-1.80	3.73
	3	2.39%	0.41%	4.40%	3.34	0.57	6.15
Gale to violent storms	0	3.13%	1.08%	5.22%	4.37	1.51	7.30
	1	3.49%	1.45%	5.58%	4.88	2.03	7.80
	2	0.25%	-1.75%	2.28%	0.34	-2.45	3.19
	3	1.86%	-0.16%	3.92%	2.60	-0.22	5.48
Hurricanes	0	7.14%	-0.78%	15.69%	9.99	-1.09	21.96
	1	0.73%	-6.96%	9.06%	1.02	-9.74	12.68
	2	2.03%	-5.65%	10.35%	2.85	-7.91	14.48
	3	4.34%	-3.35%	12.64%	6.07	-4.69	17.68

© 2026 Spriggs R et al. JAMA Network Open.

eTable 5. Percentage change in psychoactive drug-related death rates per one day increase in monthly tropical cyclone exposure by age group, cyclone strength, and lag time. Lag time was measured in months after tropical cyclone exposure. For each estimate, the corresponding change in monthly deaths per 1,000,000 population (DPM) and its 95% Bayesian credible interval are also reported. This Table accompanies Figure 3 from the main manuscript.

Age group	Cyclone strength	Lag (months)	Percentage change	95% Bayesian Credible Interval [CrI]		Additional DPM	DPM 95% Bayesian CrI	
15-29 years	All cyclones	0	9.67%	3.84%	15.85%	11.72	4.64	19.19
		1	1.71%	-3.86%	7.61%	2.08	-4.67	9.22
		2	4.82%	-0.88%	10.84%	5.83	-1.06	13.13
		3	0.89%	-4.68%	6.78%	1.08	-5.66	8.21
	Gale to violent storms	0	7.26%	1.37%	13.50%	8.79	1.66	16.34
		1	1.21%	-4.47%	7.23%	1.46	-5.41	8.75
		2	3.98%	-1.82%	10.11%	4.82	-2.20	12.24
		3	1.17%	-4.53%	7.21%	1.42	-5.49	8.73
	Hurricanes	0	30.05%	6.43%	58.92%	36.39	7.78	71.34
		1	2.18%	-18.78%	28.55%	2.64	-22.73	34.56
		2	1.65%	-18.52%	26.82%	2.00	-22.42	32.47
		3	-13.53%	-32.09%	10.11%	-16.38	-38.85	12.24
30-44 years	All cyclones	0	7.05%	3.39%	10.85%	9.62	4.62	14.80
		1	7.16%	3.49%	10.96%	9.77	4.76	14.95
		2	2.13%	-1.42%	5.82%	2.91	-1.94	7.93
		3	4.99%	1.35%	8.76%	6.80	1.84	11.94
	Gale to violent storms	0	6.59%	2.84%	10.46%	8.98	3.88	14.27
		1	6.80%	3.06%	10.68%	9.28	4.17	14.57
		2	2.48%	-1.16%	6.26%	3.39	-1.59	8.54
		3	4.28%	0.57%	8.13%	5.84	0.78	11.08
	Hurricanes	0	6.04%	-8.09%	22.34%	8.24	-11.04	30.48

© 2026 Spriggs R et al. JAMA Network Open.

	Hurricanes	1	2.88%	-10.90%	18.78%	3.92	-14.86	25.61
		2	-9.45%	-22.29%	5.50%	-12.89	-30.40	7.51
		3	6.20%	-7.62%	22.07%	8.45	-10.39	30.10
45-59 years	All cyclones	0	3.21%	0.02%	6.51%	4.43	0.03	8.97
		1	3.42%	0.24%	6.71%	4.71	0.33	9.23
		2	0.53%	-2.58%	3.74%	0.73	-3.55	5.15
		3	2.55%	-0.59%	5.80%	3.51	-0.82	7.98
	Gale to violent storms	0	2.69%	-0.57%	6.06%	3.70	-0.79	8.34
		1	3.80%	0.54%	7.17%	5.23	0.74	9.87
		2	-0.26%	-3.44%	3.01%	-0.36	-4.73	4.15
		3	1.68%	-1.53%	4.99%	2.31	-2.10	6.87
	Hurricanes	0	3.58%	-8.62%	17.42%	4.93	-11.87	23.98
		1	-9.53%	-20.90%	3.48%	-13.12	-28.78	4.79
		2	7.49%	-4.93%	21.52%	10.31	-6.78	29.63
		3	10.14%	-2.35%	24.24%	13.97	-3.24	33.37
60+ years	All cyclones	0	-1.49%	-5.69%	2.90%	-1.99	-7.62	3.88
		1	3.37%	-0.92%	7.84%	4.50	-1.23	10.49
		2	-0.37%	-4.52%	3.96%	-0.49	-6.05	5.30
		3	3.94%	-0.31%	8.36%	5.27	-0.42	11.19
	Gale to violent storms	0	-2.03%	-6.34%	2.47%	-2.72	-8.48	3.30
		1	2.23%	-2.14%	6.80%	2.99	-2.86	9.09
		2	-0.91%	-5.15%	3.53%	-1.21	-6.89	4.72
		3	3.67%	-0.68%	8.21%	4.91	-0.91	10.99
	Hurricanes	0	3.08%	-12.93%	22.05%	4.13	-17.30	29.50
		1	15.30%	-1.70%	35.25%	20.48	-2.28	47.17
		2	6.07%	-10.11%	25.18%	8.13	-13.53	33.69
		3	4.47%	-11.36%	23.13%	5.98	-15.20	30.94

© 2026 Spriggs R et al. JAMA Network Open.

eTable 6. Posterior mean differences in log-rate ratios across demographic and social disadvantage groups following tropical cyclone exposure. The bolded results are those clear of the null value, indicating a statistically significant difference.

Comparison type	Group A	Group B	Cyclone strength	Lag (months)	Mean difference	95% Bayesian Credible Interval [CrI]	
Sex	male	female	All cyclones	0	-0.03	-0.07	0.02
	male	female		1	-0.03	-0.07	0.02
	male	female		2	-0.03	-0.07	0.01
	male	female		3	-0.03	-0.07	0.02
	male	female	Gale to violent storms	0	-0.08	-0.23	0.09
	male	female		1	-0.07	-0.24	0.09
	male	female		2	-0.07	-0.24	0.09
	male	female		3	-0.07	-0.24	0.09
	male	female	Hurricanes	0	-0.03	-0.07	0.01
	male	female		1	-0.03	-0.07	0.01
	male	female		2	-0.03	-0.07	0.01
	male	female		3	-0.03	-0.07	0.01
Age	15–29 years	30–44 years	All cyclones	0	0.02	-0.04	0.09
	15–29 years	45–59 years		0	0.06	0.00	0.12
	**15–29 years**	**60+ years**		**0**	**0.11**	**0.04**	**0.18**
	30–44 years	45–59 years		0	0.04	-0.01	0.08
	**30–44 years**	**60+ years**		**0**	**0.08**	**0.03**	**0.14**
	45–59 years	60+ years		0	0.05	-0.01	0.10
	15–29 years	30–44 years		1	0.02	-0.04	0.09
	15–29 years	45–59 years		1	0.06	0.00	0.13
	**15–29 years**	**60+ years**		**1**	**0.11**	**0.04**	**0.18**
	30–44 years	45–59 years		1	0.04	-0.01	0.08
	**30–44 years**	**60+ years**		**1**	**0.08**	**0.03**	**0.14**
	45–59 years	60+ years		1	0.05	-0.01	0.10
	15–29 years	30–44 years		2	0.02	-0.04	0.09
	15–29 years	45–59 years		2	0.06	0.00	0.12

© 2026 Spriggs R et al. JAMA Network Open.

Age	**15–29 years**	**60+ years**	All cyclones	**2**	**0.11**	**0.04**	**0.18**
	30–44 years	45–59 years		2	0.04	-0.01	0.08
	**30–44 years**	**60+ years**		**2**	**0.08**	**0.03**	**0.14**
	45–59 years	60+ years		2	0.05	-0.01	0.10
	15–29 years	30–44 years		3	0.02	-0.04	0.09
	15–29 years	45–59 years		3	0.06	0.00	0.12
	**15–29 years**	**60+ years**		**3**	**0.11**	**0.04**	**0.18**
	30–44 years	45–59 years		3	0.04	-0.01	0.08
	**30–44 years**	**60+ years**		**3**	**0.08**	**0.03**	**0.14**
	45–59 years	60+ years		3	0.05	-0.01	0.10
	15–29 years	30–44 years	Gale to violent storms	0	0.01	-0.06	0.07
	15–29 years	45–59 years		0	0.04	-0.02	0.11
	**15–29 years**	**60+ years**		**0**	**0.09**	**0.02**	**0.16**
	30–44 years	45–59 years		0	0.04	-0.01	0.09
	**30–44 years**	**60+ years**		**0**	**0.09**	**0.03**	**0.14**
	45–59 years	60+ years		0	0.05	-0.01	0.10
	15–29 years	30–44 years		1	0.01	-0.06	0.07
	15–29 years	45–59 years		1	0.04	-0.02	0.11
	**15–29 years**	**60+ years**		**1**	**0.09**	**0.02**	**0.17**
	30–44 years	45–59 years		1	0.04	-0.01	0.09
	**30–44 years**	**60+ years**		**1**	**0.08**	**0.03**	**0.14**
	45–59 years	60+ years		1	0.05	-0.01	0.10
	15–29 years	30–44 years		2	0.01	-0.06	0.08
	15–29 years	45–59 years		2	0.04	-0.02	0.11
	**15–29 years**	**60+ years**		**2**	**0.09**	**0.02**	**0.16**
	30–44 years	45–59 years		2	0.04	-0.01	0.09
	**30–44 years**	**60+ years**		**2**	**0.08**	**0.03**	**0.14**
	45–59 years	60+ years		2	0.05	-0.01	0.10
	15–29 years	30–44 years		3	0.01	-0.06	0.07
	15–29 years	45–59 years		3	0.04	-0.02	0.11
	**15–29 years**	**60+ years**		**3**	**0.09**	**0.02**	**0.16**
	30–44 years	45–59 years		3	0.04	-0.01	0.08

© 2026 Spriggs R et al. JAMA Network Open.

Age	**30–44 years**	**60+ years**	Gale to violent storms	**3**	**0.08**	**0.03**	**0.14**
	45–59 years	60+ years		3	0.05	-0.01	0.10
	15–29 years	30–44 years	Hurricanes	0	0.20	-0.04	0.45
	15–29 years	45–59 years		0	0.23	-0.01	0.46
	15–29 years	60+ years		0	0.23	-0.04	0.49
	30–44 years	45–59 years		0	0.03	-0.16	0.21
	30–44 years	60+ years		0	0.03	-0.20	0.25
	45–59 years	60+ years		0	0.01	-0.20	0.21
	15–29 years	30–44 years		1	0.20	-0.05	0.44
	15–29 years	45–59 years		1	0.23	-0.01	0.46
	15–29 years	60+ years		1	0.23	-0.02	0.49
	30–44 years	45–59 years		1	0.03	-0.17	0.21
	30–44 years	60+ years		1	0.03	-0.19	0.26
	45–59 years	60+ years		1	0.01	-0.21	0.21
	15–29 years	30–44 years		2	0.21	-0.03	0.44
	15–29 years	45–59 years		2	0.23	-0.01	0.46
	15–29 years	60+ years		2	0.23	-0.03	0.49
	30–44 years	45–59 years		2	0.02	-0.17	0.21
	30–44 years	60+ years		2	0.03	-0.19	0.25
	45–59 years	60+ years		2	0.00	-0.21	0.21
	15–29 years	30–44 years		3	0.21	-0.04	0.46
	15–29 years	45–59 years		3	0.23	-0.01	0.47
	15–29 years	60+ years		3	0.23	-0.03	0.50
	30–44 years	45–59 years		3	0.02	-0.16	0.22
	30–44 years	60+ years		3	0.03	-0.19	0.25
	45–59 years	60+ years		3	0.01	-0.20	0.21
Social disadvantage	Low poverty - Low minority	Low poverty - High minority	All cyclones	0	0.06	0.00	0.13
	**Low poverty - Low minority**	**High poverty - Low minority**		**0**	**0.25**	**0.08**	**0.41**

© 2026 Spriggs R et al. JAMA Network Open.

Social disadvantage	**Low poverty - Low minority**	**High poverty - High minority**	All cyclones	**0**	**0.10**	**0.03**	**0.17**
	**Low poverty - High minority**	**High poverty - Low minority**		**0**	**0.18**	**0.03**	**0.34**
	Low poverty - High minority	High poverty - High minority		0	0.04	-0.01	0.09
	High poverty - Low minority	High poverty - High minority		0	-0.15	-0.31	0.02
	Low poverty - Low minority	Low poverty - High minority		1	0.06	0.00	0.13
	**Low poverty - Low minority**	**High poverty - Low minority**		**1**	**0.25**	**0.09**	**0.42**
	**Low poverty - Low minority**	**High poverty - High minority**		**1**	**0.10**	**0.03**	**0.17**
	**Low poverty - High minority**	**High poverty - Low minority**		**1**	**0.19**	**0.03**	**0.35**
	Low poverty - High minority	High poverty - High minority		1	0.04	-0.01	0.09
	High poverty - Low minority	High poverty - High minority		1	-0.15	-0.30	0.01
	Low poverty - Low minority	Low poverty - High minority		2	0.06	0.00	0.13
	**Low poverty - Low minority**	**High poverty - Low minority**		**2**	**0.25**	**0.08**	**0.42**
	**Low poverty - Low minority**	**High poverty - High minority**		**2**	**0.10**	**0.03**	**0.17**
	**Low poverty - High minority**	**High poverty - Low minority**		**2**	**0.18**	**0.03**	**0.34**
	Low poverty - High minority	High poverty - High minority		2	0.04	-0.01	0.09
	High poverty - Low minority	High poverty - High minority		2	-0.14	-0.30	0.02

© 2026 Spriggs R et al. JAMA Network Open.

Social disadvantage	Low poverty - Low minority	Low poverty - High minority	All cyclones	3	0.06	0.00	0.13
	**Low poverty - Low minority**	**High poverty - Low minority**		**3**	**0.25**	**0.09**	**0.41**
	**Low poverty - Low minority**	**High poverty - High minority**		**3**	**0.10**	**0.03**	**0.17**
	**Low poverty - High minority**	**High poverty - Low minority**		**3**	**0.18**	**0.02**	**0.34**
	Low poverty - High minority	High poverty - High minority		3	0.04	-0.01	0.09
	High poverty - Low minority	High poverty - High minority		3	-0.15	-0.31	0.02
	Low poverty - Low minority	Low poverty - High minority	Gale to violent storms	0	0.05	-0.02	0.12
	Low poverty - Low minority	High poverty - Low minority		0	0.15	-0.02	0.32
	Low poverty - Low minority	High poverty - High minority		0	0.06	-0.01	0.14
	Low poverty - High minority	High poverty - Low minority		0	0.10	-0.06	0.27
	Low poverty - High minority	High poverty - High minority		0	0.02	-0.04	0.07
	High poverty - Low minority	High poverty - High minority		0	-0.09	-0.26	0.08
	Low poverty - Low minority	Low poverty - High minority		1	0.05	-0.02	0.11
	Low poverty - Low minority	High poverty - Low minority		1	0.15	-0.03	0.32
	Low poverty - Low minority	High poverty - High minority		1	0.06	-0.01	0.13
	Low poverty - High minority	High poverty - Low minority		1	0.10	-0.07	0.27

© 2026 Spriggs R et al. JAMA Network Open.

Social disadvantage	Low poverty - High minority	High poverty - High minority	Gale to violent storms	1	0.02	-0.04	0.07
	High poverty - Low minority	High poverty - High minority		1	-0.09	-0.26	0.09
	Low poverty - Low minority	Low poverty - High minority		2	0.05	-0.02	0.11
	Low poverty - Low minority	High poverty - Low minority		2	0.15	-0.02	0.33
	Low poverty - Low minority	High poverty - High minority		2	0.06	-0.01	0.13
	Low poverty - High minority	High poverty - Low minority		2	0.10	-0.07	0.27
	Low poverty - High minority	High poverty - High minority		2	0.02	-0.04	0.07
	High poverty - Low minority	High poverty - High minority		2	-0.09	-0.26	0.08
	Low poverty - Low minority	Low poverty - High minority		3	0.05	-0.02	0.11
	Low poverty - Low minority	High poverty - Low minority		3	0.15	-0.02	0.32
	Low poverty - Low minority	High poverty - High minority		3	0.06	-0.01	0.14
	Low poverty - High minority	High poverty - Low minority		3	0.10	-0.06	0.27
	Low poverty - High minority	High poverty - High minority		3	0.01	-0.04	0.07
	High poverty - Low minority	High poverty - High minority		3	-0.09	-0.26	0.08
	Low poverty - Low minority	Low poverty - High minority	Hurricanes	0	0.03	-0.14	0.20
	Low poverty - Low minority	High poverty - Low minority		0	0.16	-0.59	0.89

© 2026 Spriggs R et al. JAMA Network Open.

Social disadvantage	**Low poverty - Low minority**	**High poverty - High minority**	Hurricanes	**0**	**0.59**	**0.22**	**0.95**
	Low poverty - High minority	High poverty - Low minority		0	0.14	-0.61	0.87
	**Low poverty - High minority**	**High poverty - High minority**		**0**	**0.56**	**0.19**	**0.93**
	High poverty - Low minority	High poverty - High minority		0	0.42	-0.38	1.22
	Low poverty - Low minority	Low poverty - High minority		1	0.03	-0.14	0.20
	Low poverty - Low minority	High poverty - Low minority		1	0.17	-0.58	0.89
	**Low poverty - Low minority**	**High poverty - High minority**		**1**	**0.59**	**0.20**	**0.97**
	Low poverty - High minority	High poverty - Low minority		1	0.14	-0.60	0.85
	**Low poverty - High minority**	**High poverty - High minority**		**1**	**0.55**	**0.17**	**0.93**
	High poverty - Low minority	High poverty - High minority		1	0.42	-0.40	1.22
	Low poverty - Low minority	Low poverty - High minority		2	0.03	-0.14	0.20
	Low poverty - Low minority	High poverty - Low minority		2	0.16	-0.57	0.90
	**Low poverty - Low minority**	**High poverty - High minority**		**2**	**0.58**	**0.21**	**0.97**
	Low poverty - High minority	High poverty - Low minority		2	0.14	-0.58	0.86
	**Low poverty - High minority**	**High poverty - High minority**		**2**	**0.56**	**0.18**	**0.92**
	High poverty - Low minority	High poverty - High minority		2	0.42	-0.40	1.22

© 2026 Spriggs R et al. JAMA Network Open.

Social disadvantage	Low poverty - Low minority	Low poverty - High minority	Hurricanes	3	0.03	-0.14	0.20
	Low poverty - Low minority	High poverty - Low minority		3	0.16	-0.58	0.90
	**Low poverty - Low minority**	**High poverty - High minority**		**3**	**0.59**	**0.21**	**0.95**
	Low poverty - High minority	High poverty - Low minority		3	0.14	-0.59	0.86
	**Low poverty - High minority**	**High poverty - High minority**		**3**	**0.55**	**0.18**	**0.93**
	High poverty - Low minority	High poverty - High minority		3	0.42	-0.41	1.21

© 2026 Spriggs R et al. JAMA Network Open.

eTable 7. Percentage change in psychoactive drug-related death rates per one day increase in monthly tropical cyclone exposure by sex, cyclone strength, and lag time. Lag time was measured in months after tropical cyclone exposure. For each estimate, the corresponding change in monthly deaths per 1,000,000 population (DPM) and its 95% Bayesian credible interval are also reported. This Table accompanies Figure 4 from the main manuscript.

Sex	Cyclone strength	Lag (months)	Percentage change	95% Bayesian Credible Interval [CrI]		Additional DPM	DPM 95% Bayesian CrI	
Females	All cyclones	0	6.19%	2.54%	9.97%	7.71	3.17	12.42
		1	3.37%	-0.20%	7.07%	4.20	-0.25	8.80
		2	0.33%	-3.15%	3.94%	0.41	-3.93	4.91
		3	4.21%	0.64%	7.91%	5.24	0.79	9.85
	Gale to violent storms	0	5.22%	1.51%	9.08%	6.51	1.88	11.31
		1	2.61%	-1.03%	6.39%	3.25	-1.28	7.95
		2	0.03%	-3.53%	3.73%	0.04	-4.40	4.65
		3	3.06%	-0.57%	6.84%	3.82	-0.72	8.51
	Hurricanes	0	12.88%	-1.56%	29.42%	16.04	-1.94	36.65
		1	7.41%	-6.56%	23.47%	9.23	-8.17	29.23
		2	-0.70%	-13.86%	14.48%	-0.87	-17.26	18.03
		3	12.93%	-1.18%	29.05%	16.11	-1.45	36.17
Males	All cyclones	0	2.92%	0.55%	5.34%	4.29	0.81	7.86
		1	4.20%	1.82%	6.63%	6.17	2.67	9.75
		2	1.11%	-1.21%	3.51%	1.65	-1.77	5.16
		3	2.04%	-0.30%	4.44%	3.00	-0.44	6.52
	Gale to violent storms	0	2.31%	-0.10%	4.79%	3.40	-0.15	7.05
		1	4.16%	1.72%	6.65%	6.12	2.54	9.78
		2	0.64%	-1.74%	3.07%	0.94	-2.56	4.52
		3	1.76%	-0.63%	4.21%	2.59	-0.93	6.19
	Hurricanes	0	4.85%	-4.37%	14.96%	7.13	-6.43	22.00

© 2026 Spriggs R et al. JAMA Network Open.

	Hurricanes	1	-2.49%	-11.41%	7.33%	-3.66	-16.78	10.77
		2	2.92%	-6.24%	12.97%	4.30	-9.17	19.08
		3	0.78%	-8.14%	10.57%	1.15	-11.97	15.54

© 2026 Spriggs R et al. JAMA Network Open.

eTable 8. Percentage change in psychoactive drug-related death rates per one day increase in monthly tropical cyclone exposure by county-level social disadvantage, cyclone strength, and lag time. Lag time was measured in months after tropical cyclone exposure. For each estimate, the corresponding change in monthly deaths per 1,000,000 population (DPM) and its 95% Bayesian credible interval are also reported. This Table accompanies Figure 5 from the main manuscript.

Social disadvantage	Cyclone strength	Lag (months)	Percentage change	95% Bayesian Credible Interval [CrI]		Additional DPM	DPM 95% Bayesian CrI	
Low poverty-Low minority	All cyclones	0	13.05%	6.79%	19.68%	22.82	11.87	34.41
		1	6.20%	0.29%	12.45%	10.83	0.50	21.77
		2	8.80%	2.69%	15.29%	15.39	4.70	26.73
		3	10.78%	4.68%	17.23%	18.84	8.18	30.12
	Gale to violent storms	0	9.58%	3.23%	16.32%	16.75	5.65	28.53
		1	7.07%	0.89%	13.63%	12.36	1.55	23.84
		2	9.01%	2.70%	15.72%	15.76	4.71	27.49
		3	13.06%	6.71%	19.80%	22.84	11.72	34.62
	Hurricanes	0	17.12%	4.16%	31.70%	29.93	7.27	55.42
		1	3.04%	-8.91%	16.56%	5.32	-15.58	28.96
		2	-6.53%	-19.00%	7.86%	-11.42	-33.23	13.75
		3	-8.94%	-21.28%	5.33%	-15.63	-37.20	9.33
Low poverty-High minority	All cyclones	0	6.01%	3.02%	9.08%	7.08	3.56	10.70
		1	4.95%	1.98%	8.01%	5.84	2.33	9.44
		2	1.91%	-1.04%	4.95%	2.25	-1.22	5.83
		3	2.93%	-0.10%	6.06%	3.46	-0.12	7.14
	Gale to violent storms	0	4.57%	1.53%	7.70%	5.39	1.80	9.08
		1	3.85%	0.82%	6.97%	4.53	0.96	8.21
		2	1.00%	-2.01%	4.10%	1.18	-2.37	4.84
		3	1.95%	-1.16%	5.15%	2.30	-1.37	6.07
	Hurricanes	0	13.68%	0.49%	28.59%	16.12	0.58	33.69

© 2026 Spriggs R et al. JAMA Network Open.

	Hurricanes	1	3.28%	-8.66%	16.79%	3.87	-10.21	19.78
		2	2.78%	-8.78%	15.82%	3.28	-10.35	18.64
		3	13.13%	0.27%	27.65%	15.47	0.31	32.58
High poverty-Low minority	All cyclones	0	-11.87%	-24.59%	3.01%	-19.16	-39.71	4.86
		1	-9.58%	-22.04%	4.88%	-15.47	-35.59	7.87
		2	-13.85%	-26.27%	0.67%	-22.36	-42.42	1.08
		3	-7.57%	-20.44%	7.57%	-12.22	-33.00	11.92
	Gale to violent storms	0	-5.76%	-19.99%	11.00%	-9.30	-32.28	17.77
		1	-5.65%	-19.54%	10.63%	-9.13	-31.54	17.16
		2	-13.28%	-26.19%	1.89%	-21.44	-42.29	3.06
		3	-9.41%	-22.59%	6.02%	-15.19	-36.48	9.72
	Hurricanes	0	-0.69%	-52.28%	106.91%	-1.11	-84.42	172.61
		1	20.63%	-27.44%	100.71%	33.32	-44.30	162.61
		2	-38.74%	-72.18%	34.95%	-62.55	-116.55	56.44
		3	-19.08%	-59.38%	61.25%	-30.81	-95.87	98.89
High poverty-High minority	All cyclones	0	2.08%	-2.21%	6.55%	2.98	-3.17	9.40
		1	2.11%	-2.22%	6.64%	3.03	-3.19	9.52
		2	0.10%	-4.20%	4.58%	0.14	-6.02	6.58
		3	1.26%	-3.01%	5.73%	1.81	-4.32	8.22
	Gale to violent storms	0	3.00%	-1.37%	7.57%	4.31	-1.97	10.86
		1	1.70%	-2.68%	6.28%	2.44	-3.85	9.01
		2	-0.02%	-4.37%	4.53%	-0.02	-6.27	6.50
		3	1.12%	-3.22%	5.64%	1.60	-4.61	8.09
	Hurricanes	0	-34.77%	-54.29%	-6.91%	-49.88	-77.89	-9.91
		1	6.59%	-22.73%	47.02%	9.46	-32.61	67.46
		2	-21.87%	-46.20%	13.47%	-31.37	-66.28	19.33
		3	0.84%	-27.60%	40.45%	1.20	-39.60	58.03

© 2026 Spriggs R et al. JAMA Network Open.

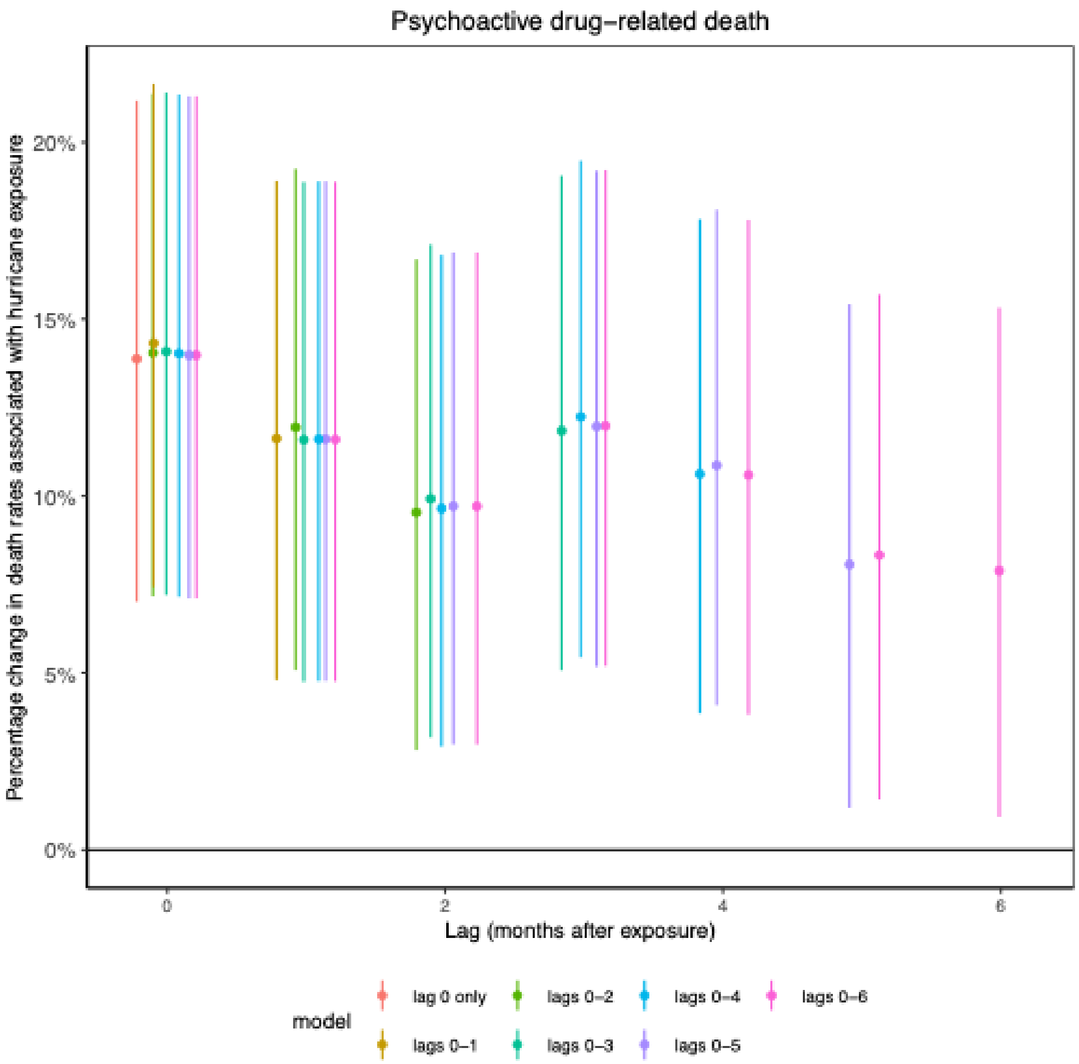

eFigure 1. Sensitivity analysis for model lag specifications.

© 2026 Spriggs R et al. JAMA Network Open.

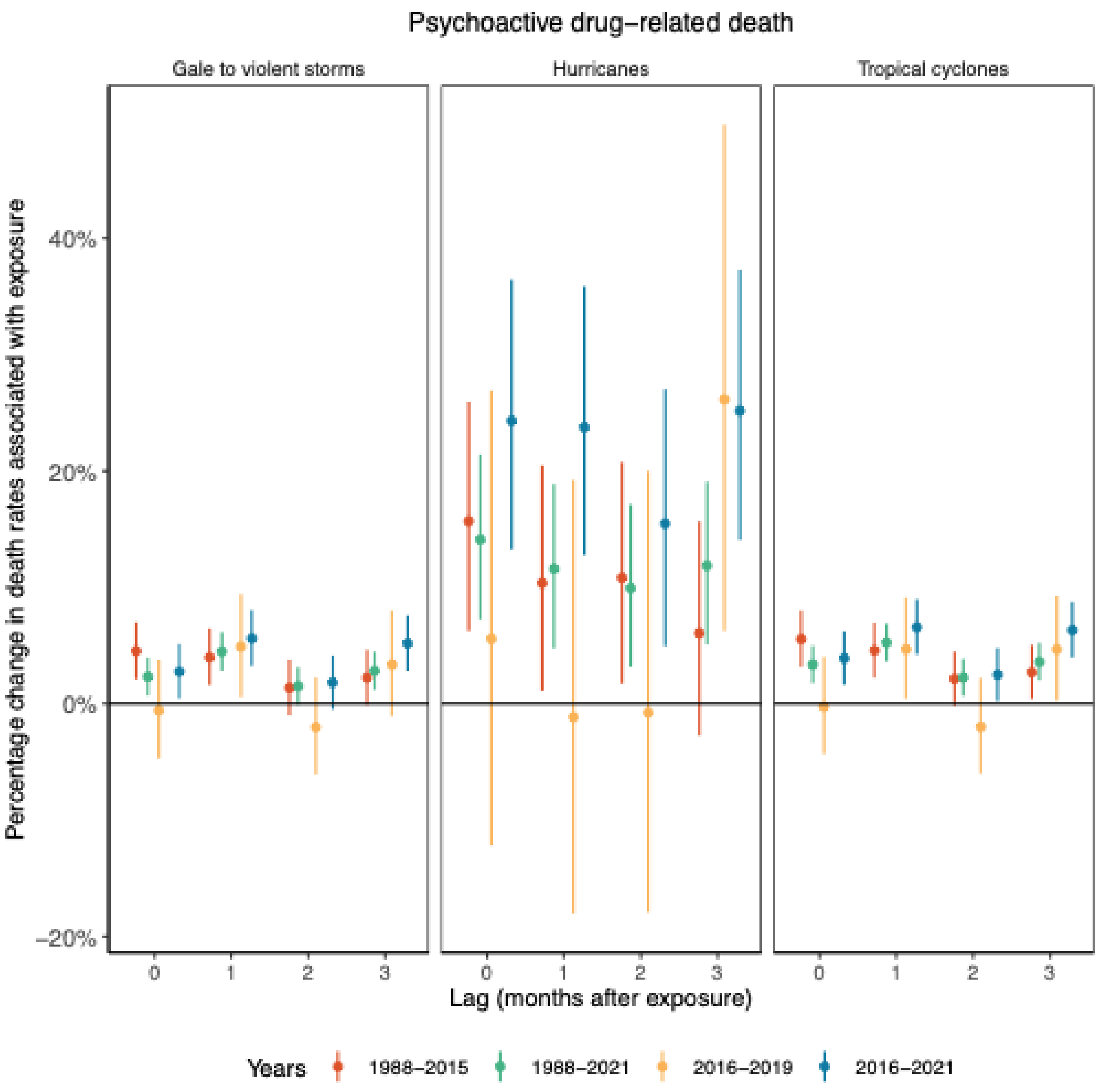

eFigure 2. Sensitivity analysis for time periods to be used in piecewise modeling.

© 2026 Spriggs R et al. JAMA Network Open.

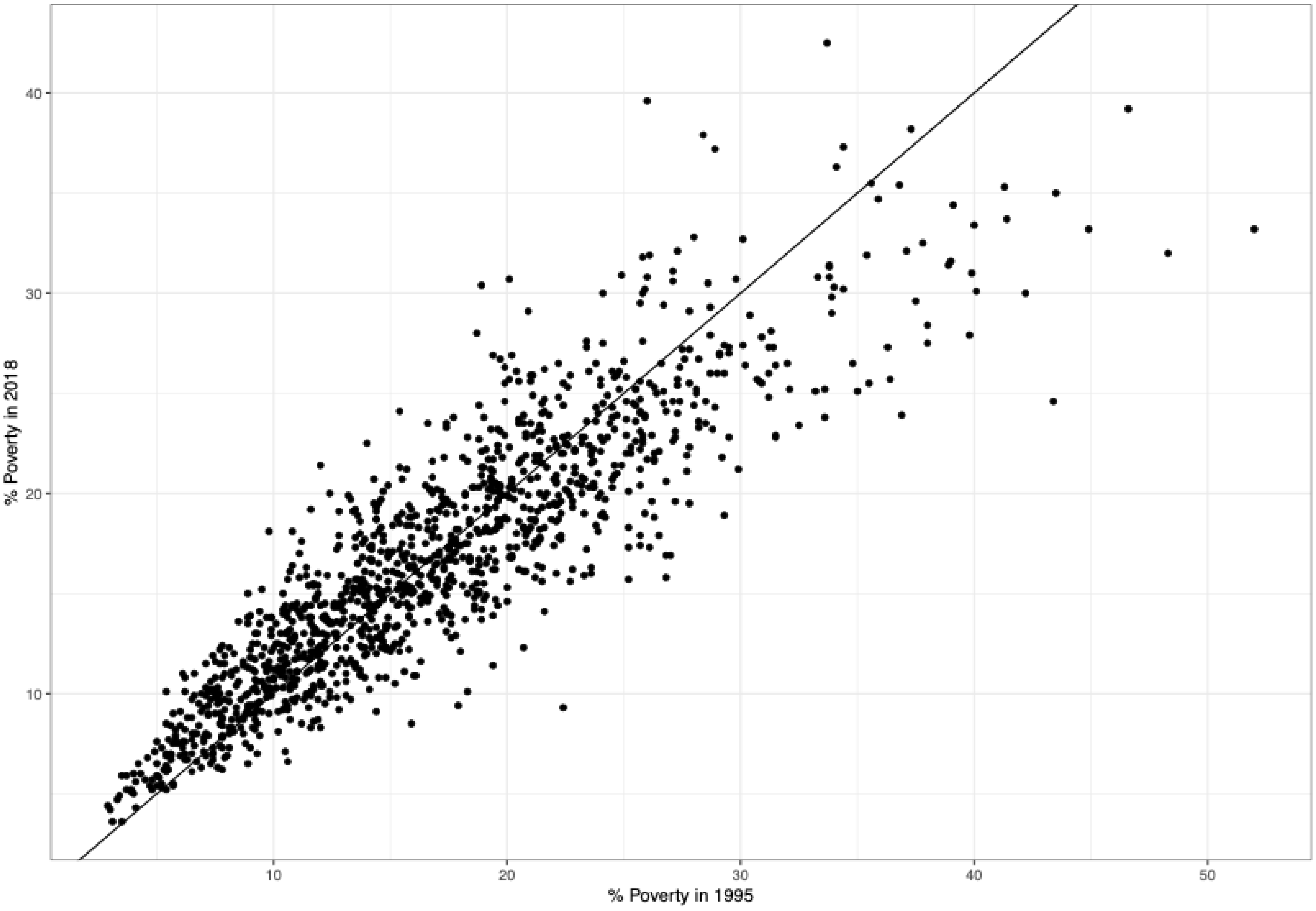

eFigure 3. Comparison of median percentage of residents in poverty for 1995 and 2018 for included U.S. counties (n=1,258).

© 2026 Spriggs R et al. JAMA Network Open.

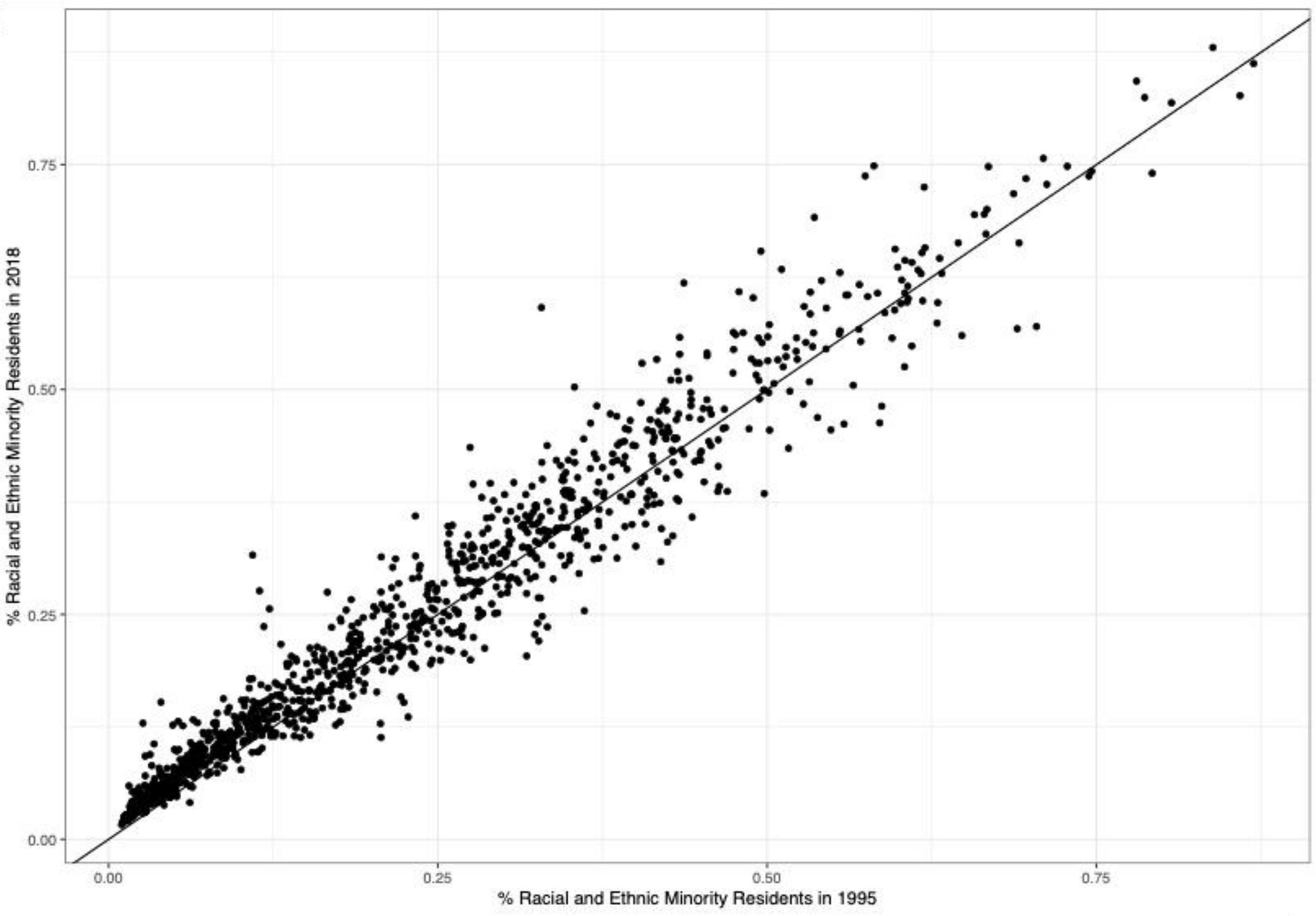

eFigure 4. Comparison of median percentage of racial and ethnic minority residents for 1995 and 2018 for included U.S. counties with racial composition data (n=1,250).

© 2026 Spriggs R et al. JAMA Network Open.

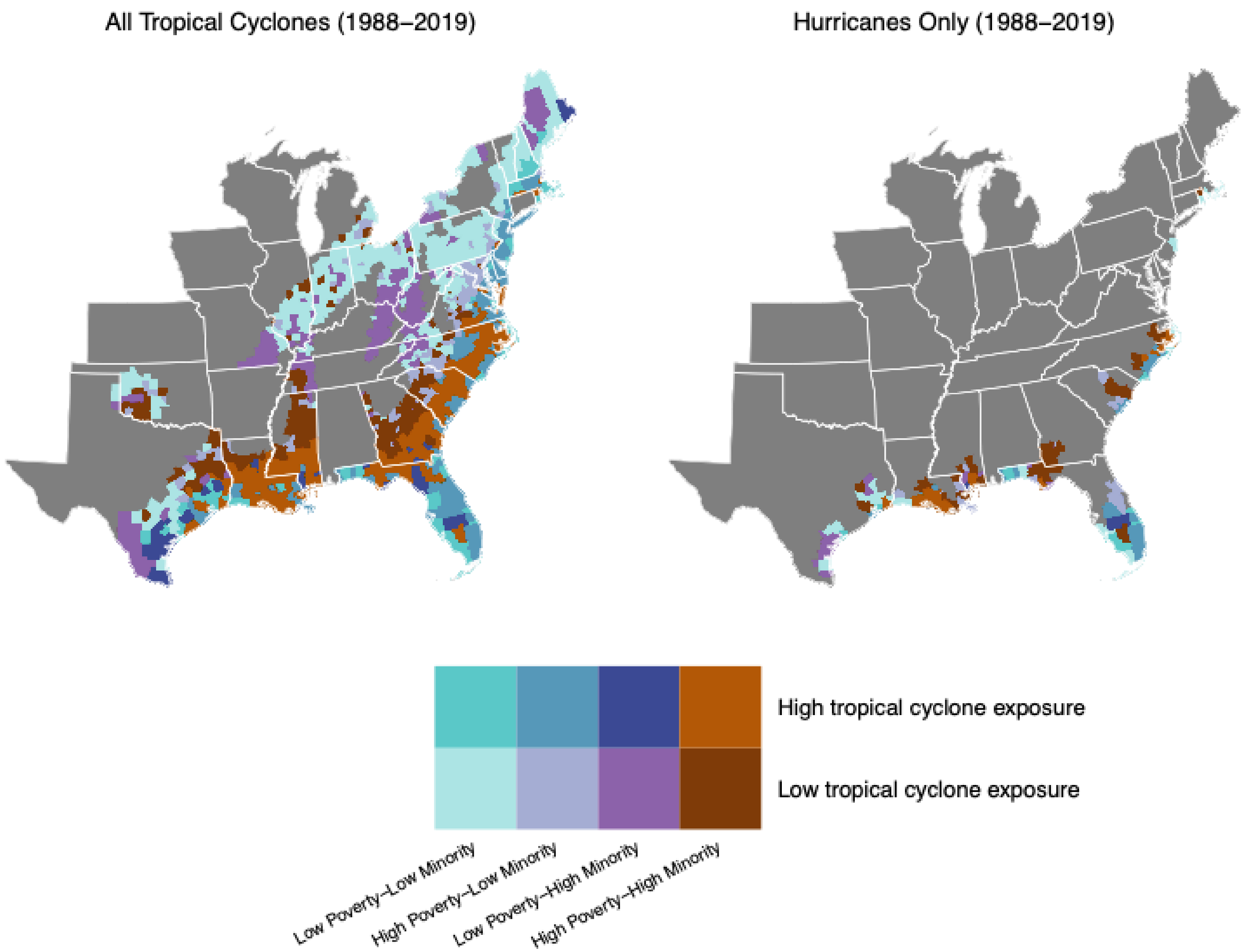

eFigure 5. Bivariate map of county-level social disadvantage and tropical cyclone exposure in U.S counties exposed to at least one tropical cyclone during 1988-2019, for all tropical cyclone exposures and for hurricane exposures only. High tropical cyclone exposure indicates exposure greater than or equal to the mean of any cyclone type (1.5 hurricane days; 4 gale to violent storm days).

© 2026 Spriggs R et al. JAMA Network Open.

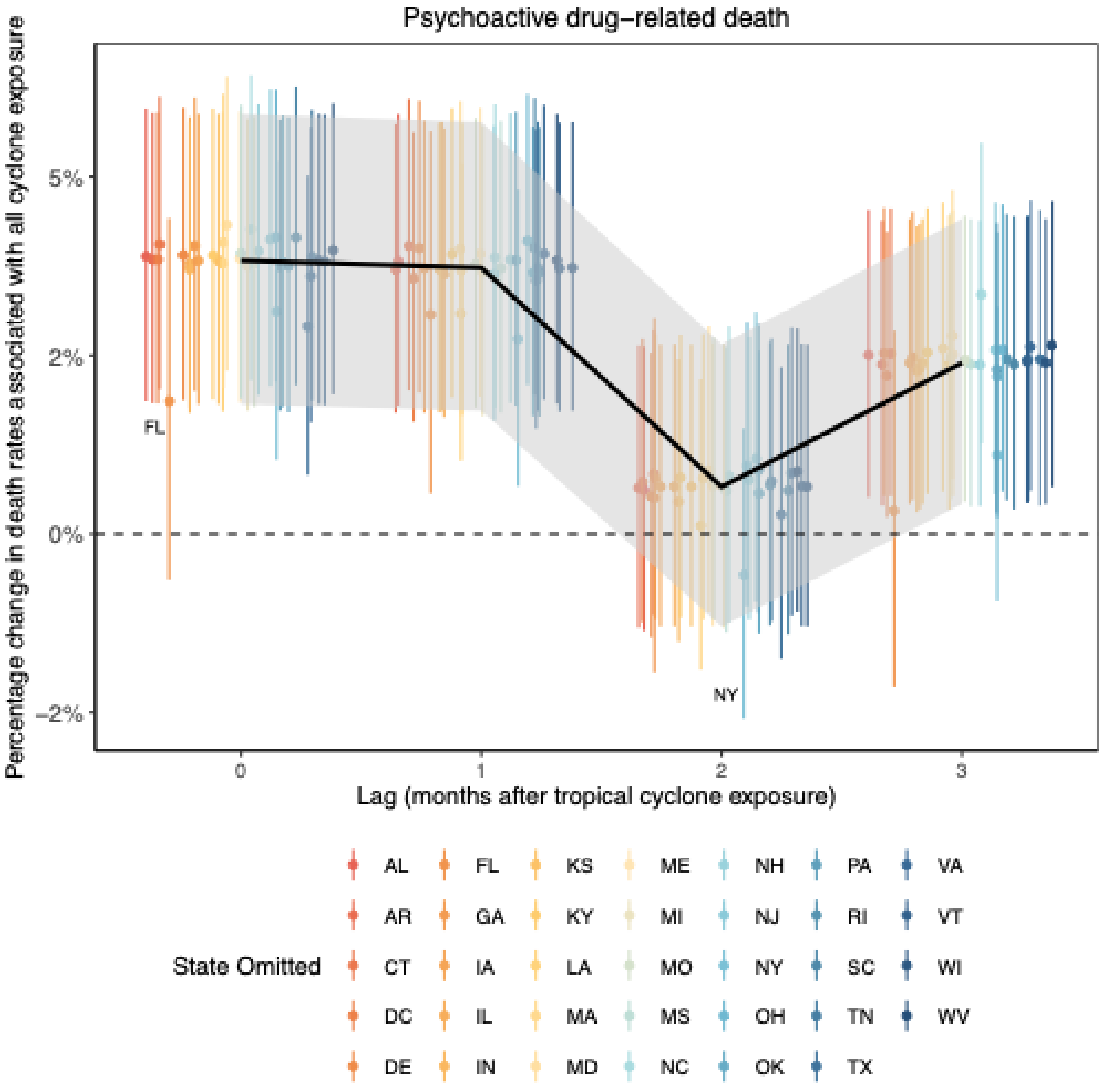

eFigure 6. Leave-one-out sensitivity analysis to assess individual U.S. state influence on the estimated national association (n=34).

© 2026 Spriggs R et al. JAMA Network Open.

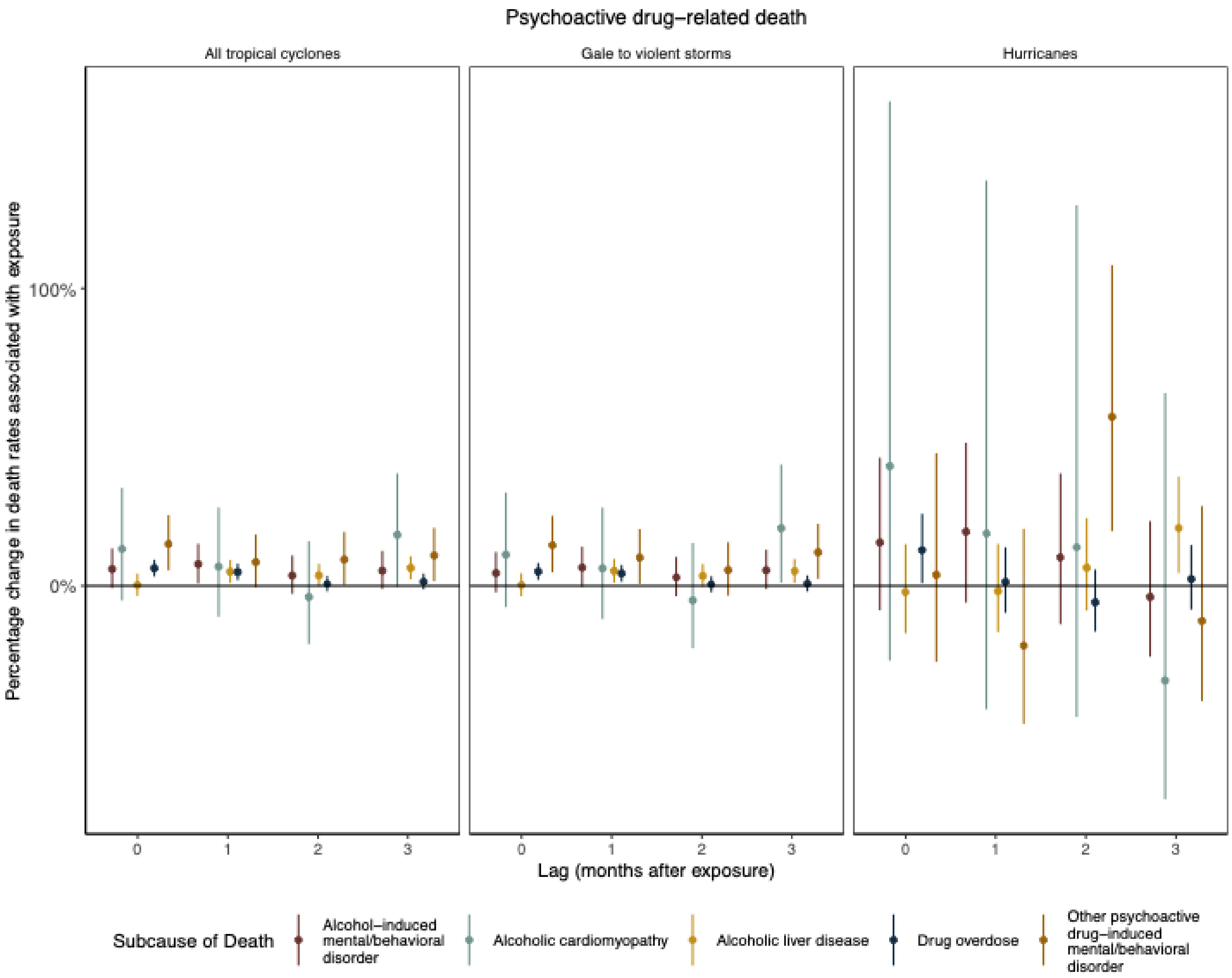

eFigure 7. Subcause sensitivity analysis to compare the estimated association across death causes classified as more acute (e.g., drug overdoses) versus more chronic (e.g., alcoholic liver disease).

© 2026 Spriggs R et al. JAMA Network Open.

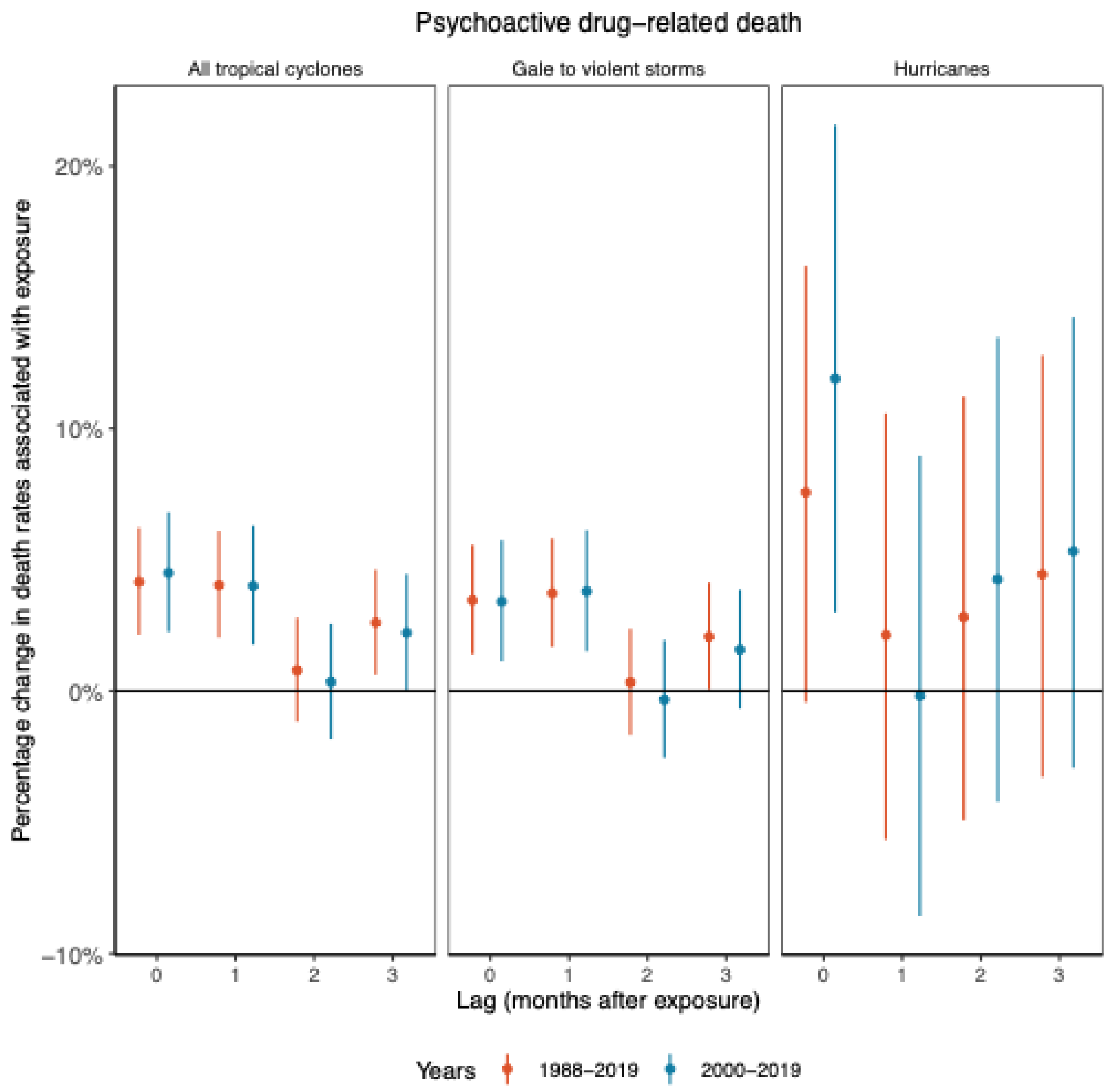

eFigure 8. Sensitivity analysis to assess whether the association changed significantly between ICD-versions 9 and 10, comparing 1988-2019 to 2000-2019.

© 2026 Spriggs R et al. JAMA Network Open.

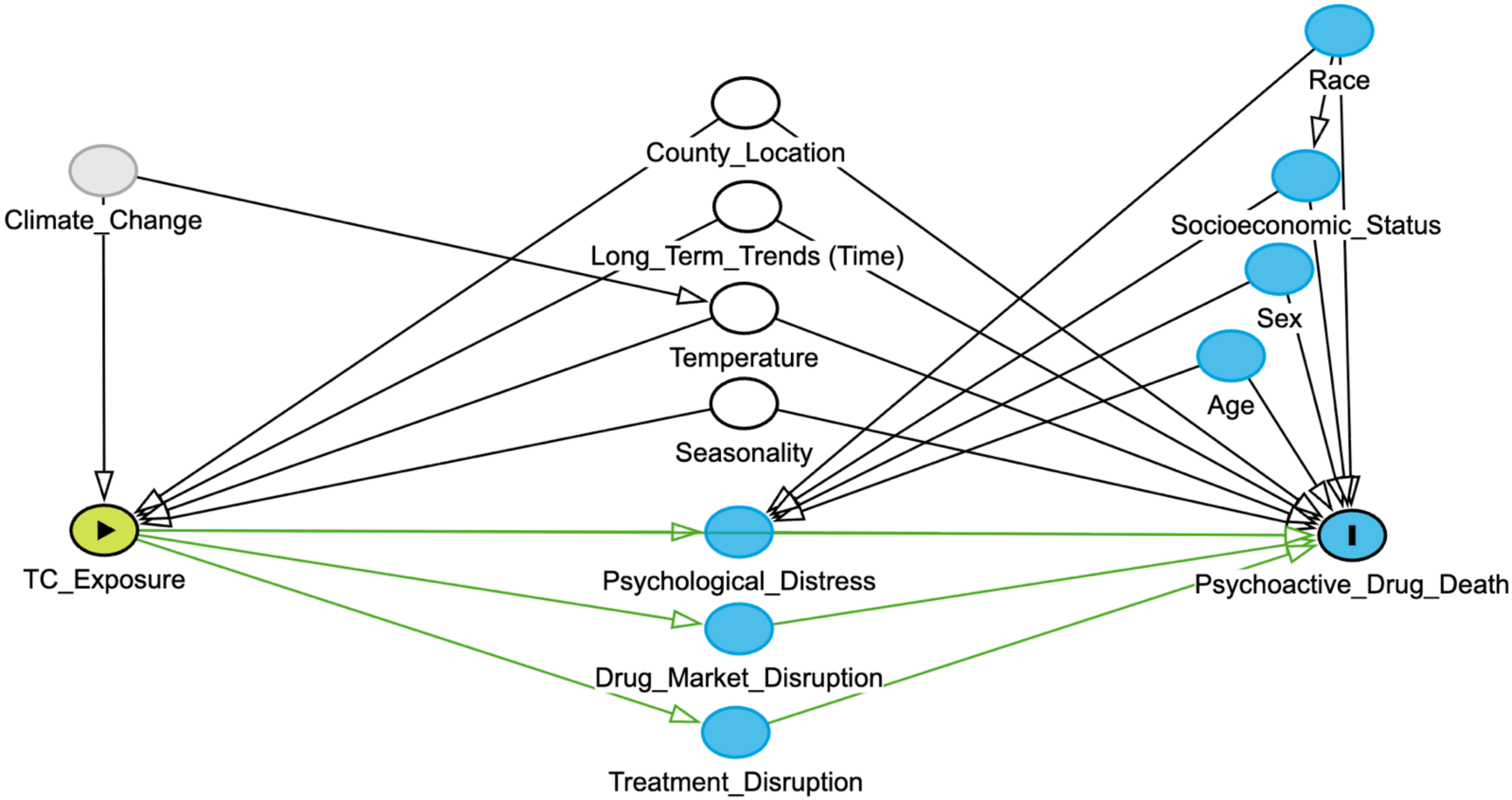

eFigure 9. Directed acyclic graph depicting the epidemiologic relationship between county-level tropical cyclone exposure and county-level psychoactive drug-related death rates. Race, socioeconomic status, sex, and age are potential effect measure modifiers, and therefore we stratified our models by age group, sex, and social disadvantage (intersection of poverty and race).

© 2026 Spriggs R et al. JAMA Network Open.
