# Supplementary material for: Tropical Cyclone Exposure and Psychoactive Drug–Related Death Rates
Source: JAMA Netw Open. 2026 Feb 20;9(2):e2560183. doi: 10.1001/jamanetworkopen.2025.60183 (PMC12924108; doi:10.1001/jamanetworkopen.2025.60183)
Supplement: Supplement 2. — Data Sharing Statement [file jamanetwopen-e2560183-s002.pdf]

Data Sharing Statement

Spriggs. Tropical Cyclone Exposure and Psychoactive Drug–Related Death Rates. *JAMA Netw Open*. Published February 20, 2026. doi:10.1001/jamanetworkopen.2025.60183

Data
Data available: Yes
Data types: Data (not involving human participants)
How to access data: Data will be available at the SPARK Lab NYC GitHub repository: https://github.com/sparklabnyc
When available: With publication

Supporting Documents
Document types: Statistical/analytic code
How to access documents: All code will be available at the SPARK Lab NYC GitHub repository: https://github.com/sparklabnyc
When available: With publication

Additional Information
Who can access the data: Anyone requesting the data
Types of analyses: For any purpose
Mechanisms of data availability: Without investigator support
